# Pregnant Women’s Perceptions of the Risks and Benefits of Disclosure During Web-Based Mental Health E-Screening Versus Paper-Based Screening: Randomized Controlled Trial

**DOI:** 10.2196/mental.6888

**Published:** 2017-10-20

**Authors:** Dawn Kingston, Anne Biringer, Sander Veldhuyzen van Zanten, Rebecca Giallo, Sarah McDonald, Glenda MacQueen, Lydia Vermeyden, Marie-Paule Austin

**Affiliations:** ^1^ University of Calgary Calgary, AB Canada; ^2^ University of Toronto Toronto, ON Canada; ^3^ University of Alberta Edmonton, AB Canada; ^4^ Murdoch Children's Research Institute Victoria Australia; ^5^ McMaster University Hamilton, ON Canada; ^6^ University of New South Wales Sydney Australia

**Keywords:** pregnancy, mental health, screening, prenatal care, computers

## Abstract

**Background:**

Pregnant women’s perceptions of the risks and benefits during mental health screening impact their willingness to disclose concerns. Early research in violence screening suggests that such perceptions may vary by mode of screening, whereby women view the anonymity of e-screening as less risky than other approaches. Understanding whether mode of screening influences perceptions of risk and benefit of disclosure is important in screening implementation.

**Objective:**

The objective of this randomized controlled trial was to compare the perceptions of pregnant women randomized to a Web-based screening intervention group and a paper-based screening control group on the level of risk and benefit they perceive in disclosing mental health concerns to their prenatal care provider. A secondary objective was to identify factors associated with women’s perceptions of risk and benefit of disclosure.

**Methods:**

Pregnant women recruited from maternity clinics, hospitals, and prenatal classes were computer-randomized to a fully automated Web-based e-screening intervention group or a paper-based control. The intervention group completed the Antenatal Psychosocial Health Assessment and the Edinburgh Postnatal Depression Scale on a computer tablet, whereas the control group completed them on paper. The primary outcome was women’s perceptions of the risk and benefits of mental health screening using the Disclosure Expectations Scale (DES). A completer analysis was conducted. Statistical significance was set at *P*<.05. We used *t* tests to compare the means of the risk and benefit subscales between groups.

**Results:**

Of the 675 eligible women approached, 636 (94.2%) agreed to participate and were randomized to the intervention (n=305) and control (n=331) groups. There were no significant baseline differences between groups. The mode of screening was not associated with either perceived risk or benefit of screening. There were no differences in groups in the mean scores of the risk and benefit of disclosure subscales. Over three-quarters of women in both intervention and control groups perceived that mental health screening was beneficial. However, 43.1% (272/631) of women in both groups reported feeling very, moderately, or somewhat vulnerable during mental health screening. We found that women of low income, those treated previously for depression or anxiety, and those pregnant with their first child were more likely to perceive greater risk. However, these associations were very small.

**Conclusions:**

Pregnant women in both the e-screening and paper-based screening groups perceived benefit and risk of disclosure similarly, suggesting that providers can implement the mode of screening that is most ideal for their clinical setting. Regardless of the mode of screening, a substantial number of women reported feeling vulnerable during mental health screening, highlighting the importance of the need to reduce women’s vulnerability throughout the screening process with strategies such as addressing women’s concerns, explaining the rationale for screening, and discussing how results will be used.

**Trial Registration:**

Clinicaltrials.gov NCT01899534; https://clinicaltrials.gov/ct2/show/NCT01899534 (Archived by WebCite at http://www.webcitation.org/6tRKtGC4M)

## Introduction

### Background

Recent studies reveal new evidence that untreated prenatal depression persists through the first 4 to 5 years postnatally, impacting child socioemotional and cognitive development [1-4]. Such evidence has been used to support recommendations for routine prenatal and postnatal mental health screening by international guidelines from the United Kingdom [5], Australia [6], and the United States [7,8], prompting major shifts in global perinatal mental health care. However, whereas the need for universal screening is clear, guidance surrounding its implementation is sparse.

One of the main considerations in implementation of routine perinatal mental health screening is the need for it to target the substantial, well-documented barriers to screening that have been reported by both women and perinatal providers [9-11]. For instance, a recent systematic review noted that even in universal screening programs comprising screening, algorithmic decision support, and direct referrals to psychiatry, depression tool screening scores were documented in only 39% of the visits [9,12]. Other studies have reported that barriers differ at each stage of perinatal mental health care (screening, referral, and treatment) [9,13], and targeting such barriers directly is the most effective approach for improving women’s access to mental health treatment [9,13]. In evaluating the implementation of routine screening in outpatient obstetrics clinics at Massachusetts General Hospital (Boston, Massachusetts), investigators concluded that “efforts that are aimed at decreasing barriers to the detection, assessment, and referral of women for depression screening both before and after delivery can lead to high levels of mental health care use among women who screen positive.” [14].

E-screening with accompanying computer-based algorithmic recommendations for treatment has potential to lessen the significant barriers that women and providers report surrounding screening and referral. Women and providers consistently report the need for support in recognizing perinatal depression and anxiety, and both feel challenged by time constraints and their discomfort in mental health discussions [9,13,15]. Providers describe the need for clear integration of screening within clinic processes and infrastructure, an easy-to-use standardized screen, and systems that link patients readily to referrals [9,13]. Threaded through all of these concerns are women’s perceptions about the risk versus the benefit of mental health screening.

Systematic reviews have suggested that women perceive risk in perinatal mental health screening, and guideline developers (including the Canadian Task Force on Preventive Health Care screening for depression) [16] have used that risk argument as a basis for not recommending routine mental health screening. However, few studies have generated strong empirical evidence on this subject [17]. Even more importantly, with the advent of novel mental health e-technologies, few studies have examined whether such perceptions vary by the mode of screening. For instance, whereas women cite risks of screening such as potentially being judged by a provider, feeling dismissed, or finding providers unsupportive, a significant implementation question is whether e-screening has potential to reduce such perceptions. On the basis of research by Renker et al [18,19] on computerized prenatal interpersonal violence screening in a demographically diverse sample of over 500 women and their reviews, e-screening may provide an anonymous venue that enables women to view the risks of screening as less daunting and the benefits more appealing [18,20-22]. Understanding whether e-screening impacts pregnant women’s perceived risks of perinatal mental health screening warrants further exploration.

### Objectives

The objective of this study was to compare pregnant women’s perception of risk and benefit of disclosure of mental health concerns based on whether they were randomized to e-screening or paper-based screening. A secondary objective was to identify factors associated with women’s perceptions of risk and benefit associated with disclosure during mental health screening.

## Methods

### Study Design

**Figure 1:**
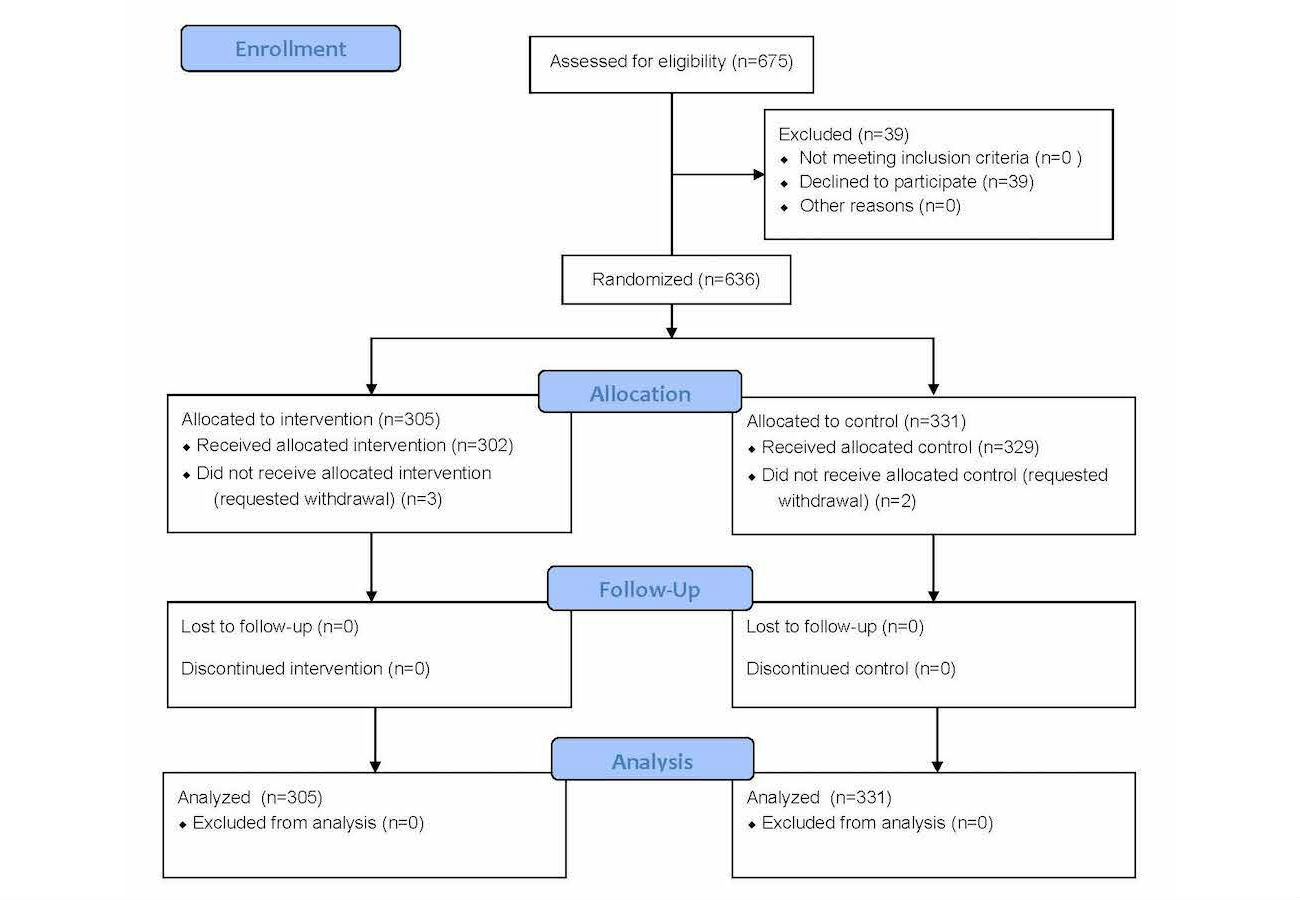
Consort flow diagram.

The study is a parallel-group, randomized controlled trial (RCT) (Figure 1). The methods have been previously published [23,24]. Approval for this study was granted by the Human Research Ethics Board at the University of Alberta.

### Eligibility Criteria

Pregnant women were eligible for this trial if they were (1) able to speak or read English, (2) willing to be randomized to e-screening, and (3) willing to participate in a follow-up diagnostic interview within 1 week of recruitment. Because the Web-based screening tool was intended to be completed unassisted, it was designed for use by women with varying levels of computer literacy.

### Setting and Recruitment

Setting and recruitment details have been published previously [23,24]. In brief, women were recruited from community-based family physician–led maternity clinics, a high-risk antenatal unit in a tertiary care center, and hospital-based prenatal classes in Edmonton, Alberta. The recruitment strategy aimed to include participants with diverse demographic and obstetrical characteristics. Trained research assistants used a standardized script to invite women to participate in the study. Once women completed the consent electronically on a computer tablet, the computer program designed by the Women’s and Children’s Health Research Institute automatically randomized them (1:1) to the intervention or control group. Thus, the research assistant was blinded to group allocation. Full details on consent procedures are found in the trial protocol [23].

### Description of E-Screening Intervention and Control Groups

The intervention is described comprehensively in the protocol (with accompanying screenshots), as are details of the Antenatal Psychosocial Health Assessment (ALPHA) [25-27] and the Edinburgh Postnatal Depression Scale (EPDS) [23]. Women randomized to the intervention group completed a full Web-based assessment with questions on psychosocial risk (ALPHA) [26,27] and current depression symptoms (EPDS) [28]. Women in the control group completed paper-based versions of the same screening tools (ALPHA and EPDS). Both groups completed the screening tools on a single occasion (recruitment).

### Procedures

The details of the questionnaires and their development are described in the protocol [23] and the first trial paper [24]. Following consent and computer randomization, women in the intervention group completed the Web-based e-screening version of the ALPHA and EPDS on a computer tablet. They then proceeded to complete the Web-based baseline questionnaire. Women in the control group completed the Web-based consent on the tablet; thereafter, they were given the paper-based versions of the ALPHA and EPDS. Once finished, they returned to the tablet to complete the Web-based baseline questionnaire. One week after recruitment, women in both groups were telephoned by a trained research assistant (blinded to group allocation) to complete a Mini-International Neuropsychiatric Interview (MINI, Version 6.0.0) [29]. No data were stored on the tablets. Upon submission, survey data were sent to a secure server housed in the Faculty of Medicine and Dentistry at the University of Alberta.

### Safety Protocol

Women who met criteria for a mood or anxiety disorder on the MINI or scored 13 or more on the EPDS were referred by the research assistant to the hospital-based reproductive mental health.

### Sample Size

Because no data were available to guide estimation of a minimal clinically important difference in true cases detected through e-screening, we used a CI approach [30]. We based the sample size calculation on 85% of women with a score of 4 to 8 on the risk subscale of the Disclosure Expectations Scale (DES) and 85% of women with a score of 16 to 20 on the utility subscale of the DES. Using a margin of error of 0.05 and 25% estimated loss to follow-up, we calculated that 261 women per group (N=542) were required[23]). At a final sample size of 636, the study was sufficiently powered to detect differences in the outcomes between groups if they exist.

### Measurement of Outcomes

We measured women’s views of the risk and benefits of e-screening using the 8-item DES. The DES comprises 2 subscales, the risk subscale (items 1, 2, 4, and 5) and the utility subscale (items 3, 6, 7, and 8), designed to identify the perceived risks and benefits of psychological care. Convergent validity of the subscales has been demonstrated with other measures of self-disclosure, as well as psychological distress and intention to seek mental health care [31]. Instructions preceding the DES asked women to consider each question within the context of discussing mental health problems with their prenatal care provider. The risk subscale assesses the level of risk and consequences women perceive in self-disclosing mental health concerns and is based on the notion that the “potential dangers of opening up to another person may seem to some individuals worse than their actual problem” [31]. The utility subscale measures the perceived value of disclosure. Participants responded to each item on a 5-point Likert scale from “very” to “not at all.” The individual scale items are given with their sample distributions in Multimedia Appendix 1.

### Analysis

Because there was a little data missing, we conducted a completer analysis (vs intention-to-treat analysis). Baseline differences of the groups were assessed using frequencies (95% CIs) and means (standard deviations [SD]) and compared using independent *t* tests (means) and chi-square tests (%) to assess the effectiveness of randomization. Statistical significance for all analyses and final models was set at *P*<.05. We used chi-square tests to compare proportions of women in each group responding to the subscale items.

Before the multivariable analysis, we conducted bivariate analyses to identify independent factors that were significantly associated with each of the outcomes at *P*<.20, estimating unadjusted odds ratios and their 95% CIs. Those variables were entered in the final multivariable models simultaneously, where *P*<.05 defined factors that were significantly associated with the outcomes in the final models.

## Results

### Sample Characteristics

Of the 675 eligible women approached from August 2013 to January 2015, 636 agreed to participate (participation rate: 94.2%, 636/675) and were randomized to the intervention (n=305) and control (n=331) groups. A total of 5 women withdrew from the study following group allocation: 3 in the intervention group and 2 in the control group (see Figure 1). There were no statistically significant differences at baseline between the two groups.

Table 1 shows that the majority of pregnant women were between 25 and 34 years of age, partnered, white, had incomes of Can $80,000 or more, had at least some postsecondary education and were pregnant with their first child. One-quarter of participants had been diagnosed and treated for a mental health concern before recruitment. The majority of women were comfortable using laptops, computer tablets, and smartphones. Missing data were less than 3.0% (19/636) for all variables, with the majority having less than 1.5% (10/636); thus, data imputation was not used.

### Primary and Secondary Objectives

#### Primary Objectives

##### Perceived Risk and Benefit of Disclosure: Description of Items of the Risk and Utility Subscales

There were no significant differences between groups on any of the items of the risk or benefit subscales of the DES (Multimedia Appendix 1). In terms of risk, the item with the most endorsements was “How vulnerable would you feel if you disclosed something very personal to your doctor or nurse that you have never told anyone before,” with 42.4% (128/302) of women in the e-screening group and 43.8% (144/329) in the paper-based group indicating disclosure of a mental health concern would make them feel somewhat, moderately, or very vulnerable (Multimedia Appendix 1). This was followed by women endorsing that they would perceive disclosure as somewhat or moderately or very “risky” (e-screening 34.4% [104/302]; paper 35.3% [116/329]), “worrisome” (e-screening 29.5% [89/302]; paper 32.5% [107/329]), and “difficult” (e-screening 22.2% [67/302]; paper 21.0% [69/329]) (Multimedia Appendix 1).

From a benefits perspective, the majority of women in both groups felt they would get a useful response from their provider if they disclosed their concerns (e-screening 81.1% [245/302]; paper 83.9% [276/329]), and it would be beneficial to do so (e-screening 83.1% [251/302]; paper 81.5% [268/329]). Additionally, 76.8% (485/631) of women felt that it would be helpful to talk to their provider about a mental health problem (e-screening 76.2% [230/302]; paper 77.5% [255/329]), and it would feel better to have the opportunity to discuss their feelings of anxiety or depression with them (e-screening 70.9% [214/302]; paper 77.5% [255/329]).

**Table 1:** Sample characteristics (N=636).

Characteristics		Full sample (N=636^a^)	Paper-based screening group (n=331^a^)	E-screening group (n=305^a^)	*P* value^b^
**Recruitment site, n (%)**					
	Community-based clinic	423 (67.8)	224 (70.0)	199 (65.5)	.47
	High-risk antenatal unit	70 (11.2)	34 (10.6)	36 (11.8)	
Prenatal class, n (%)		131 (21.0)	62 (19.4)	69 (22.7)	
**Age, n (%)**					
	<25 years	88 (13.9)	50 (15.2)	38 (12.5)	.51
	25-34 years	459 (72.2)	233 (70.6)	226 (74.6)	
	35+	86 (13.6)	47 (14.2)	39 (12.9)	
**Income, n (%)**					
	Below $40,000	97 (15.4)	52 (15.8)	45 (14.9)	.81
	$40,000-$79,999	139 (22.0)	75 (22.8)	64 (21.2)	
	$80,000 or more	395 (62.6)	202 (61.4)	193 (63.9)	
**Education, n (%)**					
	High school or less	100 (15.8)	57 (17.3)	43 (14.2)	.29
	Some postsecondary or more	531 (84.2)	272 (82.7)	259 (85.8)	
**Marital status, n (%)**					
	Unpartnered	27 (4.3)	14 (4.3)	13 (4.3)	.98
	Partnered	604 (95.7)	315 (95.7)	289 (95.7)	
**Ethnicity, n (%)**					
	Not white	169 (26.8)	91 (27.7)	78 (25.8)	.60
	white	462 (73.2)	238 (72.3)	224 (74.2)	
**Born in Canada, n (%)**					
	No	119 (18.9)	66 (20.1)	53 (17.5)	.42
	Yes	512 (81.1)	263 (79.9)	249 (82.5)	
**Ever diagnosed with depression, anxiety, or any other kind of emotional concern, n (%)**					
	Yes	164 (25.9)	86 (26.1)	78 (25.7)	.91
	No	470 (74.1)	244 (73.9)	226 (74.3)	
**Ever treated for depression, anxiety, or any other kind of emotional concern, n (%)**					
	Yes	179 (28.2)	92 (27.9)	87 (28.6)	.84
	No	455 (71.8)	238 (72.1)	217 (71.4)	
**Pregnant before, n (%)**					
	First child	426 (69.3)	213 (68.5)	213 (70.1)	.67
	Not first child	189 (30.7)	98 (31.5)	91 (29.9)	
Weeks gestation, mean (SD^c^)		9.00 (6.46)	8.61 (6.08)	9.39 (6.80)	.22
**Used fertility treatments to become pregnant, n (%)**					
	Yes	35 (5.5)	17 (5.2)	18 (5.9)	.67
	No	599 (94.5)	313 (94.8)	286 (94.1)	
**ACEs^d^** **score n (%)**					
	Score greater than or equal to 4	113 (18.0)	64 (19.5)	49 (16.3)	.31
	Score less than 4	516 (82.0)	265 (80.5)	251 (83.7)	
**I am comfortable using a computer or laptop, n (%)**					
	Very comfortable	591 (93.7)	311 (94.5)	280 (92.7)	.45
	Somewhat comfortable	36 (5.7)	17 (5.2)	19 (6.3)	
	Not very comfortable	4 (0.6)	1 (0.3)	3 (1.0)	
**I am comfortable using a computer tablet (eg, iPad), n (%)**					
	Very comfortable	530 (84.0)	280 (85.1)	250 (82.8)	.64
	Somewhat comfortable	89 (14.1)	44 (13.4)	45 (14.9)	
	Not very comfortable	12 (1.9)	5 (1.5)	7 (2.3)	
**I am comfortable using a mobile phone, n (%)**					
	Very comfortable	546 (86.5)	286 (86.9)	260 (86.1)	.32
	Somewhat comfortable	70 (11.1)	38 (11.6)	32 (10.6)	
	Not very comfortable	15 (2.4)	5 (1.5)	10 (3.3)	

^a^Some demographic data missing.

^b^Comparison of control and intervention groups: χ^2^ statistic used for variables with three or more categories; two-tailed *t* test used for variables with estimated means.

^c^SD: standard deviation.

^d^ACEs: adverse childhood experiences.

##### Perceived Risk and Benefit of Disclosure: Mean Scores of the Risk and Utility Subscales

There were no statistically significant differences between the e-screening and paper-based groups on the mean (SD) scores of the risk subscale (mean=8.51, SD=3.59 vs mean=8.57, SD=3.73) nor the utility (benefit) subscale (mean=14.11, SD=4.05 vs mean=14.17, SD=4.03) (Table 2).

#### Secondary Outcome

##### Factors Associated With Perceiving Risk in Disclosure of Prenatal Mental Health Problems

Among the twelve independent variables that we tested (including mode of screening), five variables were significantly associated with perceived risk of disclosing prenatal mental health problems: income, marital status, previously treated for depression or anxiety, born in Canada, and parity (data not shown). In the final multivariable linear regression model (Table 3), low income, being treated previously for depression or anxiety, and being pregnant with the first child were significantly associated with perceiving greater risk in disclosing mental health concerns. On the basis of the partial eta squared, the effect size for each of these variables in terms of their contributions to risk of disclosure is very small.

##### Factors Associated With Perceiving Benefit in Disclosure of Prenatal Mental Health Problems

In bivariate analyses, age (under 25 years) and nulliparity were significantly associated with the perceived benefit of disclosure based on the utility subscale of the DES. No variables were significant in the final multiple linear regression model of factors associated with pregnant women perceiving benefit in disclosing mental health problems to their prenatal care providers (Table 4).

**Table 2:** Mean scores of risk and benefit subscales of the Disclosure Expectations Scale (N=629).

Primary outcome	Overall, mean (SD)	Paper, mean (SD)	E-screening, mean (SD)	*t* statistic (degrees of freedom)	*P* value^a^
Risk score	8.54 (3.66)	8.57 (3.73)	8.51 (3.59)	0.222 (629)	.82
Benefit score	14.14 (4.03)	14.17 (4.03)	14.11 (4.05)	0.189 (629)	.85

^a^Comparison of control and intervention groups: χ^2^ statistic used for variables with three or more categories; two-tailed *t* test used for variables with estimated means.

**Table 3:** Multiple linear regression of factors associated with perceiving risk in disclosure of prenatal mental health problems.

Variable^a^	Beta (95% CI)	Standard error	Beta	*P* value	Partial eta squared^b^
Income (less than Can $40,000)	1.11 (0.25-1.98)	0.44	.11	.01	0.010
Marital status (unpartnered)	.69 (−0.77 to 2.16)	0.75	.04	.35	0.001
Treated previously for depression anxiety (treated)^c^	.84 (0.19-1.49)	0.33	.10	.01	0.010
Born in Canada (No)^c^	−.76 (−1.55 to 0.03)	0.40	−.08	.06	0.006
Parity (first child)	.85 (0.23-1.46)	0.31	.11	.007	0.012

^a^Independent variables with *P*<.20 were entered simultaneously into the final model, including income, marital status, previously treated for depression or anxiety, born in Canada, and parity. The supplementary table of the univariate analysis is available from the corresponding author.

^b^On the basis of guidelines [32], a partial eta squared of >0.01 is a small effect size, >0.06 is medium, and >0.14 is large.

^c”^Diagnosed and treated previously for depression or anxiety” were highly correlated and could not be entered into the same model (Pearson *r*=.85). Similarly, “born in Canada” and “ethnicity” were highly correlated (Pearson *r*=.60) and not entered together.

**Table 4:** Multiple linear regression of factors associated with pregnant women perceiving benefit in disclosure of prenatal mental health problems.

Variable^a^	B (95% CI)	Standard error	Beta	*P* value	Partial eta squared^b^
Age (under 25 years)	−.77 (−1.68 to 0.15)	0.47	−.07	.10	0.004
Parity (first child)	.21 (−0.49 to 0.90)	0.35	.02	.56	0.001

^a^Independent variables with *P*<.20 were entered simultaneously into the final model, including maternal age and parity. The supplementary table of the univariate analysis is available from the corresponding author.

^b^On the basis of guidelines [32], a partial eta squared of >0.01 is a small effect size, >0.06 is medium, and >0.14 is large.

## Discussion

### Interpretation

This trial adds substantially to the limited evidence on implementation of screening during the perinatal period by providing data on women’s views of the benefits and risks of disclosure of mental health concerns by mode of screening. In this study, 76.8 (485/631) of women perceived that mental health screening was beneficial. However, 21.6% (136/631) to 43.1% (272/631) of women perceived that disclosure held some degree of risk in that they viewed it as risky and worrisome, reporting that it made them feel vulnerable. There were no differences in groups in the mean scores of the risk and benefit of disclosure subscales. In multivariable linear regression analyses, we found that women of low income, those who had been treated previously for depression or anxiety, and those pregnant with their first child were more likely to perceive a greater risk in disclosing mental health concerns compared with women of higher income, who had never been treated for mental health problems, and who were multiparous. We found no factors that were associated with perceiving benefit in screening. Mode of screening (paper-based vs e-screening) was not significantly associated with either perceived risk or benefit of screening.

Overall, pregnant women perceived both paper-based and e-mental health screening to be beneficial. These findings are consistent with our cross-sectional study (N=460), where 97.6% (449/460) of pregnant women surveyed reported that they were very or somewhat comfortable with completing paper-based screening at home (92.3%, 425/460) or in a maternity clinic (90.4%, 416/460), as well as computer-based (86.0%, 395/460) screening [33]. They are also consistent with the study’s finding that 97.3% (448/460) of pregnant women were comfortable with provider-initiated screening, whereas only two-thirds were comfortable with self-initiating discussions about their mental health concerns. Others have also reported a general acceptability of routine mental health screening in Australia, following the initiation of universal prenatal screening through the National Depression Initiative [34-37] and in the United States in hospital-based [14] and regional perinatal screening programs [38].

Women’s views of the benefits of screening did not vary by mode of screening. This result indicates that the way women were screened (paper or e-screening) did not influence the value of screening that women perceived in terms of its overall benefit, usefulness, helpfulness, or contribution in making them feel better. This positive finding suggests that whatever mode of screening providers choose to implement in their clinical settings will be viewed as beneficial by women. Similarly, the nonsignificant difference in the mean scores of the risk subscale reveals that women in the paper-based and e-screening groups viewed the degree of risk of disclosure similarly. On one hand, this is positive in that the providers can be assured that the risk that women perceive is independent of the mode of screening they choose to employ in their clinical settings.

However, it is concerning that 43.1% (272/631) of women find screening a vulnerable process. Again, that a similar number of women in both groups reported some degree of vulnerability indicates that this was unrelated to the way the screening questions were delivered and more likely linked to other aspects of the screening process such as the way screening is introduced or debriefed, provider characteristics, or the provider-client relationship. Several studies have shown the importance of provider characteristics and relationships on screening, including being heard and trusting the provider [39], the ability of the provider to make a connection, being empathetic [40] and being a “good fit” (eg, we “clicked”) [13] were key aspects of successful treatment, whereas friendly, sensitive, warm, and caring attributes facilitated the screening process [41]. Conversely, negative experiences with perinatal health care providers have also been shown as detrimental to addressing perinatal depression, including women having their concerns dismissed, perceiving that their provider was inadequately prepared to assess and discuss perinatal depression, being unprepared for the process or the nature of the questions, feeling anxious and vulnerable when raising distressing histories, and seeing the screening process as intrusive [42]. Our own studies mirror these findings. We reported that women who had a relationship with their provider that fostered honesty were less likely to be deterred by potential barriers to screening [15,33,43], and those who had a sensitive and caring and interested provider were more likely to engage in screening [15,33,43]. These studies all support the conclusion that “the way in which clinicians interact with patients about depression might strongly influence patient responses” [39]. Our research has also shown that women were more likely to engage in screening if certain aspects of the process were in place, such as having an explanation about why some sensitive questions were asked, knowing what to expect if she revealed emotional struggles, being reassured that other women also have prenatal emotional problems, and knowing that talking about emotional health is a part of routine prenatal care [15].

We might have seen a difference in vulnerability by screening mode if we had included a face-to-face screening arm. For instance, qualitative studies of postpartum women have reported that face-to-face screening and discussions around treatment make women feel significantly vulnerable [44,45]. The findings of this study support the importance of the screening process as a whole, in that the mode of screening alone (e-screening vs paper) does not seem to mitigate the vulnerability that women experience during mental health screening.

Although the effect sizes were small, the findings that women of low income, those who had been treated previously for depression or anxiety, and those pregnant with their first child were more likely to perceive a greater risk in disclosing mental health concerns are important in identifying potential subgroups of women who may find screening a more vulnerable process. Given that our sample was quite demographically homogeneous, further research on the views of screening among these subgroups of women is warranted.

Of importance, this study demonstrated that mode of screening was not associated with perceived risk of screening. This finding is positive in light of how little we know about how women perceive e-screening and suggests that e-screening is a viable option for delivering mental health screening. Finally, that no subgroups of women were identified as perceiving greater or less benefit from screening suggests that all women, regardless of demographics or previous mental health history, find mental health screening beneficial. Mode of screening was also not identified as having an impact on perceived benefit, indicating that women find equal benefit from screening regardless of whether the questions are delivered on paper or tablet.

### Limitations

Our sample was quite demographically homogeneous with the majority of women being partnered and well educated, as well as being born in Canada. However, our findings suggest that some subgroups of women may perceive mental health screening as more vulnerable. Future research should explore such women’s views of mental health screening in greater depth.

### Conclusions

Women in this sample generally perceived mental health assessment as beneficial. However, a substantial number of them felt vulnerable during the screening process for mental health issues, and their perceptions were not mitigated by the mode of screening. Mode of screening was not related to women’s perceptions of the risk or benefit of screening.

## Appendix

Multimedia Appendix 1 Risks and benefits of disclosure of paper-based versus e-screening (N=631).

Multimedia Appendix 2 CONSORT‐EHEALTH checklist (V.1.6.1).

## Abbreviations

ALPHA: Antenatal Psychosocial Health Assessment
DES: Disclosure Expectations Scale
EPDS: Edinburgh Postnatal Depression Scale
MINI: Mini-International Neuropsychiatric Interview
NHMRC: National Health and Medical Research Council
RCT: randomized controlled trial
SD: standard deviation
