# Supplementary material for: Pregnant Women’s Perceptions of the Risks and Benefits of Disclosure During Web-Based Mental Health E-Screening Versus Paper-Based Screening: Randomized Controlled Trial
Source: JMIR Ment Health. 2017 Oct 20;4(4):e42. doi: 10.2196/mental.6888 (PMC5670313; doi:10.2196/mental.6888)
Supplement: Multimedia Appendix 1 [file mental_v4i4e42_app1.pdf]

Appendix 1. Risks and benefits of disclosure of paper-based versus e-screening (N=631).

Disclosure Expectations Scale items[a]		Full sample (N=631[b]) N (%)	Group 1 Paper-based screening (n=329) n (%)	Group 2 E-screening (n=302) n (%)	X^2 (degrees of freedom)	*P* value[b]
**1. How difficult would it be for you to disclose personal information to a doctor or nurse?**						
	Not at all	323 (51.2)	171 (52.0)	152 (50.3)	2.2_4	.70
	Slightly	172 (27.3)	89 (27.1)	83 (27.5)		
	Somewhat	84 (13.3)	44 (13.4)	40 (13.2)		
	Moderately	45 (7.1)	20 (6.1)	25 (8.3)		
	Very	7 (1.1)	5 (1.5)	2 (0.7)		
**2. How vulnerable would you feel if you disclosed something very personal to your doctor or nurse that you have never told anyone before?**						
	Not at all	154	76 (23.1)	78 (25.8)	2.4_4	.66

		(24.4)				
	Slightly	205 (32.5)	109 (33.1)	96 (31.8)		
	Somewhat	125 (19.8)	67 (20.4)	58 (19.2)		
	Moderately	86 (13.6)	49 (14.9)	37 (12.3)		
	Very	61 (9.7)	28 (8.5)	33 (10.9)		
**3. How beneficial would it be for you to talk about the problem with a doctor or nurse?**						
	Not at all	39 (6.2)	21 (6.4)	18 (6.0)	2.2_4	.69
	Slightly	73 (11.6)	40 (12.2)	33 (10.9)		
	Somewhat	120 (19.0)	66 (20.1)	54 (17.9)		
	Moderately	167 (26.5)	90 (27.4)	77 (25.5)		
	Very	232 (36.8)	112 (34.0)	120 (39.7)		
**4. How risky would it feel to disclose your hidden feelings to a doctor or nurse?**						
	Not at all	251 (39.8)	127 (38.6)	124 (41.1)	2.5_4	.64

	Slightly	160 (25.4)	86 (26.1)	74 (24.5)		
	Somewhat	117 (18.5)	65 (19.8)	52 (17.2)		
	Moderately	87 (13.8)	41 (12.5)	46 (15.2)		
	Very	16 (2.5)	10 (3.0)	6 (2.0)		
**5. How worried would you be about what a doctor or nurse thought if you disclosed negative emotions?**						
	Not at all	243 (38.5)	122 (37.1)	121 (40.1)	1.8_4	.77
	Slightly	192 (30.4)	100 (30.4)	92 (30.5)		
	Somewhat	113 (17.9)	65 (19.8)	48 (15.9)		
	Moderately	64 (10.1)	32 (9.7)	32 (10.6)		
	Very	19 (3.0)	10 (3.0)	9 (3.0)		
**6. How helpful would it be to you to talk about a personal problem to a doctor or nurse?**						
	Not at all	47 (7.4)	20 (6.1)	27 (8.9)	2.4_4	.66
	Slightly	99 (15.7)	54 (16.4)	45 (14.9)		

	Somewhat	147 (23.3)	79 (24.0)	68 (22.5)		
	Moderately	172 (27.3)	87 (26.4)	85 (28.1)		
	Very	166 (26.3)	89 (27.1)	77 (25.5)		
**7. Would you feel better if you talked about feelings of sadness or anxiety to a doctor or nurse?**						
	Not at all	54 (8.6)	28 (8.5)	26 (8.6)	5.3_4	.26
	Slightly	108 (17.1)	46 (14.0)	62 (20.5)		
	Somewhat	154 (24.4)	87 (26.4)	67 (22.2)		
	Moderately	171 (27.1)	91 (27.7)	80 (26.5)		
	Very	144 (22.8)	77 (23.4)	67 (22.2)		
**8. How likely would you be to get a useful response if you disclosed an emotional problem you have with a doctor or nurse?**						

	Not at all	33 (5.2)	19 (5.8)	14 (4.6)	2.7_4	.61
	Slightly	77 (12.2)	34 (10.3)	43 (14.2)		
	Somewhat	203 (32.2)	110 (33.4)	93 (30.8)		
	Moderately	176 (27.9)	92 (28.0)	84 (27.8)		
	Very	142 (22.5)	74 (22.5)	68 (22.5)		

[a]Risk subscale items include 1, 2, 4, and 5; Utility (benefit) subscale items include 3, 6, 7, and 8.

[b]Total sample missing data=9 (Group 1: Paper-based screening=6; Group 2: E-screening=3).
