# Supplementary material for: Pregnant Women’s Perceptions of the Risks and Benefits of Disclosure During Web-Based Mental Health E-Screening Versus Paper-Based Screening: Randomized Controlled Trial
Source: JMIR Ment Health. 2017 Oct 20;4(4):e42. doi: 10.2196/mental.6888 (PMC5670313; doi:10.2196/mental.6888)
Supplement: Multimedia Appendix 2 [file mental_v4i4e42_app2.pdf]

CONSORT-EHEALTH (V 1.6.1) - Submission/Publication Form

The CONSORT-EHEALTH checklist is intended for authors of randomized trials evaluating web-based and Internet-based applications/interventions, including mobile interventions, electronic games (incl multiplayer games), social media, certain telehealth applications, and other interactive and/or networked electronic applications. Some of the items (e.g. all subitems under item 5 - description of the intervention) may also be applicable for other study designs.

The goal of the CONSORT EHEALTH checklist and guideline is to be
a) a guide for reporting for authors of RCTs,
b) to form a basis for appraisal of an ehealth trial (in terms of validity)

CONSORT-EHEALTH items/subitems are MANDATORY reporting items for studies published in the Journal of Medical Internet Research and other journals / scientific societies endorsing the checklist.

Items numbered 1., 2., 3., 4a., 4b etc are original CONSORT or CONSORT-NPT (non-pharmacologic treatment) items.
Items with Roman numerals (i., ii, iii, iv etc.) are CONSORT-EHEALTH extensions/clarifications.

As the CONSORT-EHEALTH checklist is still considered in a formative stage, we would ask that you also RATE ON A SCALE OF 1-5 how important/useful you feel each item is FOR THE PURPOSE OF THE CHECKLIST and reporting guideline (optional).

Mandatory reporting items are marked with a red *.
In the textboxes, either copy & paste the relevant sections from your manuscript into this form - please include any quotes from your manuscript in QUOTATION MARKS,
or answer directly by providing additional information not in the manuscript, or elaborating on why the item was not relevant for this study.

YOUR ANSWERS WILL BE PUBLISHED AS A SUPPLEMENTARY FILE TO YOUR PUBLICATION IN JMIR AND ARE CONSIDERED PART OF YOUR PUBLICATION (IF ACCEPTED).
Please fill in these questions diligently. Information will not be copyedited, so please use proper spelling and grammar, use correct capitalization, and avoid abbreviations.

DO NOT FORGET TO SAVE AS PDF _AND_ CLICK THE SUBMIT BUTTON SO YOUR ANSWERS ARE IN OUR DATABASE !!!

Citation Suggestion (if you append the pdf as Appendix we suggest to cite this paper in the caption):
Eysenbach G, CONSORT-EHEALTH Group
CONSORT-EHEALTH: Improving and Standardizing Evaluation Reports of Web-based and Mobile Health Interventions
J Med Internet Res 2011;13(4):e126
URL: http://www.jmir.org/2011/4/e126/
doi: 10.2196/jmir.1923
PMID: 22209829

* Required

Your response exceeds the limit. Try shortening some of your answers.

Your name *

First Last

Kingston

Primary Affiliation (short), City, Country *

University of Toronto, Toronto, Canada

University of Calgary, Calgary

Your e-mail address *

abc@gmail.com

dawn.kingston@ucalgary.ca

Title of your manuscript *

Provide the (draft) title of your manuscript.

Pregnant Women's Perceptions of the Risks and Benefits of Disclosure During Web-Based Mental Health E-Screening Versus Paper-Based Screening: Randomized Controlled Trial

Article Preparation Status/Stage *

At which stage in your article preparation are you currently (at the time you fill in this form)

- ○ not submitted yet - in early draft status
- ○ not submitted yet - in late draft status, just before submission
- ○ submitted to a journal but not reviewed yet
- ○ submitted to a journal and after receiving initial reviewer comments
- ◉ submitted to a journal and accepted, but not published yet
- ○ published
- ○ Other:

Journal *

If you already know where you will submit this paper (or if it is already submitted), please provide the journal name (if it is not JMIR, provide the journal name under "other")

- ○ not submitted yet / unclear where I will submit this
- ○ Journal of Medical Internet Research (JMIR)
- ◉ Other: JMIR Mental Health

Manuscript tracking number *

If this is a JMIR submission, please provide the manuscript tracking number under "other" (The ms tracking number can be found in the submission acknowledgement email, or when you login as author in JMIR. If the paper is already published in JMIR, then the ms tracking number is the four-digit number at the end of the DOI, to be found at the bottom of each published article in JMIR)

- ○ no ms number (yet) / not (yet) submitted to / published in JMIR
- ◉ Other: #6888

TITLE AND ABSTRACT

1a) TITLE: Identification as a randomized trial in the title

1a) Does your paper address CONSORT item 1a? *

I.e does the title contain the phrase "Randomized Controlled Trial"? (if not, explain the reason under "other")

- (●) yes
- ( ) Other:

1a-i) Identify the mode of delivery in the title

Identify the mode of delivery. Preferably use “web-based” and/or “mobile” and/or “electronic game” in the title. Avoid ambiguous terms like “online”, “virtual”, “interactive”. Use “Internet-based” only if Intervention includes non-web-based Internet components (e.g. email), use “computer-based” or “electronic” only if offline products are used. Use “virtual” only in the context of “virtual reality” (3-D worlds). Use “online” only in the context of “online support groups”. Complement or substitute product names with broader terms for the class of products (such as “mobile” or “smart phone” instead of “iphone”), especially if the application runs on different platforms.

	1	2	3	4	5	
subitem not at all important	( )	( )	( )	( )	(●)	essential

Does your paper address subitem 1a-i? *

Copy and paste relevant sections from manuscript title (include quotes in quotation marks "like this" to indicate direct quotes from your manuscript), or elaborate on this item by providing additional information not in the ms, or briefly explain why the item is not applicable/relevant for your study

Web-Based Mental Health E-Screening

1a-ii) Non-web-based components or important co-interventions in title

Mention non-web-based components or important co-interventions in title, if any (e.g., “with telephone support”).

	1	2	3	4	5	
subitem not at all important	( )	( )	( )	( )	(●)	essential

Does your paper address subitem 1a-ii?

Copy and paste relevant sections from manuscript title (include quotes in quotation marks "like this" to indicate direct quotes from your manuscript), or elaborate on this item by providing additional information not in the ms, or briefly explain why the item is not applicable/relevant for your study

Versus Paper-Based Screening

1a-iii) Primary condition or target group in the title

Mention primary condition or target group in the title, if any (e.g., “for children with Type I Diabetes”) Example: A Web-based and Mobile Intervention with Telephone Support for Children with Type I Diabetes: Randomized Controlled Trial

	1	2	3	4	5	
subitem not at all important	○	○	○	○	○	essential

Does your paper address subitem 1a-iii? *

Copy and paste relevant sections from manuscript title (include quotes in quotation marks "like this" to indicate direct quotes from your manuscript), or elaborate on this item by providing additional information not in the ms, or briefly explain why the item is not applicable/relevant for your study

Pregnant Women’s

1b) ABSTRACT: Structured summary of trial design, methods, results, and conclusions

NPT extension: Description of experimental treatment, comparator, care providers, centers, and blinding status.

1b-i) Key features/functionalities/components of the intervention and comparator in the METHODS section of the ABSTRACT

Mention key features/functionalities/components of the intervention and comparator in the abstract. If possible, also mention theories and principles used for designing the site. Keep in mind the needs of systematic reviewers and indexers by including important synonyms. (Note: Only report in the abstract what the main paper is reporting. If this information is missing from the main body of text, consider adding it)

	1	2	3	4	5	
subitem not at all important	○	○	○	○	◉	essential

Does your paper address subitem 1b-i? *

Copy and paste relevant sections from the manuscript abstract (include quotes in quotation marks "like this" to indicate direct quotes from your manuscript), or elaborate on this item by providing additional information not in the ms, or briefly explain why the item is not applicable/relevant for your study

Objective: The objective of this randomized controlled trial was to compare the perceptions of pregnant women randomized to a Web-based screening intervention group and a paper-based screening control group on the level of risk and benefit they perceive in disclosing mental health concerns to their prenatal care provider. A secondary objective was to identify factors associated with women's perceptions of risk and benefit of disclosure.
Methods: Pregnant women recruited from maternity clinics, hospitals, and prenatal classes were computer-randomized to a fully automated Web-based e-screening intervention group or a paper-based control. The intervention group completed the Antenatal Psychosocial Health Assessment and the Edinburgh Postnatal Depression Scale on a computer tablet, whereas the control group completed them on paper. The primary outcome was women's perceptions of the risk and benefits of mental health screening using the Disclosure Expectations Scale (DES). A completer analysis was conducted. Statistical significance was set at P<.05. We used t tests to compare the means of the risk and benefit subscales between groups.

1b-ii) Level of human involvement in the METHODS section of the ABSTRACT

Clarify the level of human involvement in the abstract, e.g., use phrases like "fully automated" vs. "therapist/nurse/care provider/physician-assisted" (mention number and expertise of providers involved, if any). (Note: Only report in the abstract what the main paper is reporting. If this information is missing from the main body of text, consider adding it)

	1	2	3	4	5	
subitem not at all important	○	○	○	○	◉	essential

Does your paper address subitem 1b-ii?

Copy and paste relevant sections from the manuscript abstract (include quotes in quotation marks "like this" to indicate direct quotes from your manuscript), or elaborate on this item by providing additional information not in the ms, or briefly explain why the item is not applicable/relevant for your study

Pregnant women recruited from maternity clinics, hospitals, and prenatal classes were computer-randomized to a fully automated Web-based e-screening intervention group or a paper-based control. The intervention group completed the Antenatal Psychosocial Health Assessment and the Edinburgh Postnatal Depression Scale on a computer tablet, whereas the control group completed them on paper.

1b-iii) Open vs. closed, web-based (self-assessment) vs. face-to-face assessments in the METHODS section of the ABSTRACT

Mention how participants were recruited (online vs. offline), e.g., from an open access website or from a clinic or a closed online user group (closed usergroup trial), and clarify if this was a purely web-based trial, or there were face-to-face components (as part of the intervention or for assessment). Clearly say if outcomes were self-assessed through questionnaires (as common in web-based trials). Note: In traditional offline trials, an open trial (open-label trial) is a type of clinical trial in which both the researchers and participants know which treatment is being administered. To avoid confusion, use "blinded" or "unblinded" to indicated the level of blinding instead of "open", as "open" in web-based trials usually refers to "open access" (i.e. participants can self-enrol). (Note: Only report in the abstract what the main paper is reporting. If this information is missing from the main body of text, consider adding it)

	1	2	3	4	5	
subitem not at all important	○	○	○	○	◉	essential

Does your paper address subitem 1b-iii?

Copy and paste relevant sections from the manuscript abstract (include quotes in quotation marks "like this" to indicate direct quotes from your manuscript), or elaborate on this item by providing additional information not in the ms, or briefly explain why the item is not applicable/relevant for your study

Pregnant women recruited from maternity clinics, hospitals, and prenatal classes were computer-randomized to a fully automated Web-based e-screening intervention group or a paper-based control. The intervention group completed the Antenatal Psychosocial Health Assessment and the Edinburgh Postnatal Depression Scale on a computer tablet, whereas the control group completed them on paper.

1b-iv) RESULTS section in abstract must contain use data

Report number of participants enrolled/assessed in each group, the use/uptake of the intervention (e.g., attrition/adherence metrics, use over time, number of logins etc.), in addition to primary/secondary outcomes. (Note: Only report in the abstract what the main paper is reporting. If this information is missing from the main body of text, consider adding it)

	1	2	3	4	5	
subitem not at all important	○	○	○	○	●	essential

Does your paper address subitem 1b-iv?

Copy and paste relevant sections from the manuscript abstract (include quotes in quotation marks "like this" to indicate direct quotes from your manuscript), or elaborate on this item by providing additional information not in the ms, or briefly explain why the item is not applicable/relevant for your study

Of the 675 eligible women approached, 636 (94.2%) agreed to participate and were randomized to the intervention (n=305) and control (n=331) groups. There were no significant baseline differences between groups. The mode of screening was not associated with either perceived risk or benefit of screening. There were no differences in groups in the mean scores of the risk and benefit of disclosure subscales. Over three-quarters of women in both intervention and control groups perceived that mental health screening was beneficial. However, 43.1% (272/631) of women in both groups reported feeling very, moderately, or somewhat vulnerable during mental health screening. We found that women of low income, those treated previously for depression or anxiety, and those pregnant with their first child were more likely to perceive greater risk. However, these associations were very small.
The primary outcome was women's perceptions of the risk and benefits of mental health screening using the Disclosure Expectations Scale (DES). A completer analysis was conducted. Statistical significance was set at P<.05. We used t tests to compare the means of the risk and benefit subscales between groups. A secondary objective was to identify factors associated with women's perceptions of risk and benefit of disclosure.

1b-v) CONCLUSIONS/DISCUSSION in abstract for negative trials

Conclusions/Discussions in abstract for negative trials: Discuss the primary outcome - if the trial is negative (primary outcome not changed), and the intervention was not used, discuss whether negative results are attributable to lack of uptake and discuss reasons. (Note: Only report in the abstract what the main paper is reporting. If this information is missing from the main body of text, consider adding it)

1 2 3 4 5

subitem not at all important ○ ○ ○ ◉ ○ essential

Does your paper address subitem 1b-v?

Copy and paste relevant sections from the manuscript abstract (include quotes in quotation marks "like this" to indicate direct quotes from your manuscript), or elaborate on this item by providing additional information not in the ms, or briefly explain why the item is not applicable/relevant for your study

Pregnant women in both the e-screening and paper-based screening groups perceived benefit and risk of disclosure similarly, suggesting that providers can implement the mode of screening that is most ideal for their clinical setting. Regardless of the mode of screening, a substantial number of women reported feeling vulnerable during mental health screening, highlighting the importance of the need to reduce women's vulnerability throughout the screening process with strategies such as addressing women's concerns, explaining the rationale for screening, and discussing how results will be used.

INTRODUCTION

2a) In INTRODUCTION: Scientific background and explanation of rationale

2a-i) Problem and the type of system/solution

Describe the problem and the type of system/solution that is object of the study: intended as stand-alone intervention vs. incorporated in broader health care program? Intended for a particular patient population? Goals of the intervention, e.g., being more cost-effective to other interventions, replace or complement other solutions? (Note: Details about the intervention are provided in "Methods" under 5)

	1	2	3	4	5	
subitem not at all important	○	○	○	○	◉	essential

Does your paper address subitem 2a-i? *

Copy and paste relevant sections from the manuscript (include quotes in quotation marks "like this" to indicate direct quotes from your manuscript), or elaborate on this item by providing additional information not in the ms, or briefly explain why the item is not applicable/relevant for your study

One of the main considerations in implementation of routine perinatal mental health screening is the need for it to target the substantial, well-documented barriers to screening that have been reported by both women and perinatal providers [9-11].
E-screening with accompanying computer-based algorithmic recommendations for treatment has potential to lessen the significant barriers that women and providers report surrounding screening and referral. Women and providers consistently report the need for support in recognizing perinatal depression and anxiety, and both feel challenged by time constraints and their discomfort in mental health discussions [9,13,15]. Providers describe the need for clear integration of screening within clinic processes and infrastructure, an easy-to-use standardized screen, and systems that link patients readily to referrals [9,13]. E-mental health screening also has the benefit of being efficient, effective and resource-sparing.

2a-ii) Scientific background, rationale: What is known about the (type of) system

Scientific background, rationale: What is known about the (type of) system that is the object of the study (be sure to discuss the use of similar systems for other conditions/diagnoses, if appropiate), motivation for the study, i.e. what are the reasons for and what is the context for this specific study, from which stakeholder viewpoint is the study performed, potential impact of findings [2]. Briefly justify the choice of the comparator.

	1	2	3	4	5	
subitem not at all important	○	○	○	○	◉	essential

Does your paper address subitem 2a-ii? *

Copy and paste relevant sections from the manuscript (include quotes in quotation marks "like this" to indicate direct quotes from your manuscript), or elaborate on this item by providing additional information not in the ms, or briefly explain why the item is not applicable/relevant for your study

Recent studies reveal new evidence that untreated prenatal depression persists through the first 4 to 5 years postnatally, impacting child socioemotional and cognitive development [1-4]. Such evidence has been used to support recommendations for routine prenatal and postnatal mental health screening by international guidelines from the United Kingdom [5], Australia [6], and the United States [7,8], prompting major shifts in global perinatal mental health care. However, whereas the need for universal screening is clear, guidance surrounding its implementation is sparse.

2b) In INTRODUCTION: Specific objectives or hypotheses

Does your paper address CONSORT subitem 2b? *

Copy and paste relevant sections from the manuscript (include quotes in quotation marks "like this" to indicate direct quotes from your manuscript), or elaborate on this item by providing additional information not in the ms, or briefly explain why the item is not applicable/relevant for your study

The objective of this study was to compare pregnant women's perception of risk and benefit of disclosure of mental health concerns based on whether they were randomized to e-screening or paper-based screening. A secondary objective was to identify factors associated with women's perceptions of risk and benefit associated with disclosure during mental health screening.

METHODS

3a) Description of trial design (such as parallel, factorial) including allocation ratio

Does your paper address CONSORT subitem 3a? *

Copy and paste relevant sections from the manuscript (include quotes in quotation marks "like this" to indicate direct quotes from your manuscript), or elaborate on this item by providing additional information not in the ms, or briefly explain why the item is not applicable/relevant for your study

The study is a parallel-group, randomized controlled trial (RCT):
Intervention n=305 Control n=331

3b) Important changes to methods after trial commencement (such as eligibility criteria), with reasons

Does your paper address CONSORT subitem 3b? *

Copy and paste relevant sections from the manuscript (include quotes in quotation marks "like this" to indicate direct quotes from your manuscript), or elaborate on this item by providing additional information not in the ms, or briefly explain why the item is not applicable/relevant for your study

Pregnant women were eligible for this trial if they were (1) able to speak or read English, (2) willing to be randomized to e-screening, and (3) willing to participate in a follow-up diagnostic interview within 1 week of recruitment. Because the Web-based screening tool was intended to be completed unassisted, it was designed for use by women with varying levels of computer literacy.
Eligibility criteria remained unchanged.

3b-i) Bug fixes, Downtimes, Content Changes

Bug fixes, Downtimes, Content Changes: ehealth systems are often dynamic systems. A description of changes to methods therefore also includes important changes made on the intervention or comparator during the trial (e.g., major bug fixes or changes in the functionality or content) (5-iii) and other “unexpected events” that may have influenced study design such as staff changes, system failures/downtimes, etc. [2].

	1	2	3	4	5	
subitem not at all important	(•)	( )	( )	( )	( )	essential

Does your paper address subitem 3b-i?

Copy and paste relevant sections from the manuscript (include quotes in quotation marks "like this" to indicate direct quotes from your manuscript), or elaborate on this item by providing additional information not in the ms, or briefly explain why the item is not applicable/relevant for your study

N/A There were no major bug fixes or changes in the functionality of the surveys.

4a) Eligibility criteria for participants

Does your paper address CONSORT subitem 4a? *

Copy and paste relevant sections from the manuscript (include quotes in quotation marks "like this" to indicate direct quotes from your manuscript), or elaborate on this item by providing additional information not in the ms, or briefly explain why the item is not applicable/relevant for your study

The eligibility criteria for participants remained unchanged.

4a-i) Computer / Internet literacy

Computer / Internet literacy is often an implicit “de facto” eligibility criterion - this should be explicitly clarified.

	1	2	3	4	5	
subitem not at all important	○	○	○	●	○	essential

Does your paper address subitem 4a-i?

Copy and paste relevant sections from the manuscript (include quotes in quotation marks "like this" to indicate direct quotes from your manuscript), or elaborate on this item by providing additional information not in the ms, or briefly explain why the item is not applicable/relevant for your study

Because the Web-based screening tool was intended to be completed unassisted, it was designed for use by women with varying levels of computer literacy.

4a-ii) Open vs. closed, web-based vs. face-to-face assessments:

Open vs. closed, web-based vs. face-to-face assessments: Mention how participants were recruited (online vs. offline), e.g., from an open access website or from a clinic, and clarify if this was a purely web-based trial, or there were face-to-face components (as part of the intervention or for assessment), i.e., to what degree got the study team to know the participant. In online-only trials, clarify if participants were quasi-anonymous and whether having multiple identities was possible or whether technical or logistical measures (e.g., cookies, email confirmation, phone calls) were used to detect/prevent these.

1 2 3 4 5

subitem not at all important ○ ○ ◉ ○ ○ essential

Does your paper address subitem 4a-ii? *

Copy and paste relevant sections from the manuscript (include quotes in quotation marks "like this" to indicate direct quotes from your manuscript), or elaborate on this item by providing additional information not in the ms, or briefly explain why the item is not applicable/relevant for your study

Offline recruitment took place at community-based family physician–led maternity clinics, a high-risk antenatal unit in a tertiary care center, and hospital-based prenatal classes in Edmonton,

4a-iii) Information giving during recruitment

Information given during recruitment. Specify how participants were briefed for recruitment and in the informed consent procedures (e.g., publish the informed consent documentation as appendix, see also item X26), as this information may have an effect on user self-selection, user expectation and may also bias results.

	1	2	3	4	5	
subitem not at all important	○	○	◉	○	○	essential

Does your paper address subitem 4a-iii?

Copy and paste relevant sections from the manuscript (include quotes in quotation marks "like this" to indicate direct quotes from your manuscript), or elaborate on this item by providing additional information not in the ms, or briefly explain why the item is not applicable/relevant for your study

Trained research assistants used a standardized script to invite women to participate in the study. Once women completed the consent electronically on a computer tablet, the computer program designed by the Women’s and Children’s Health Research Institute automatically randomized them (1:1) to the intervention or control group. Thus, the research assistant

was blinded to group allocation

4b) Settings and locations where the data were collected

Does your paper address CONSORT subitem 4b? *

Copy and paste relevant sections from the manuscript (include quotes in quotation marks "like this" to indicate direct quotes from your manuscript), or elaborate on this item by providing additional information not in the ms, or briefly explain why the item is not applicable/relevant for your study

In brief, women were recruited from community-based family physician–led maternity clinics, a high-risk antenatal unit in a tertiary care center, and hospital-based prenatal classes in Edmonton, Alberta.

4b-i) Report if outcomes were (self-)assessed through online questionnaires

Clearly report if outcomes were (self-)assessed through online questionnaires (as common in web-based trials) or otherwise.

	1	2	3	4	5	
subitem not at all important	○	○	○	◉	○	essential

Does your paper address subitem 4b-i? *

Copy and paste relevant sections from the manuscript (include quotes in quotation marks "like this" to indicate direct quotes from your manuscript), or elaborate on this item by providing additional information not in the ms, or briefly explain why the item is not applicable/relevant for your study

Women randomized to the intervention group completed a full Web-based assessment with questions on psychosocial risk (ALPHA) [26,27] and current depression symptoms (EPDS) [28]. Women in the control group completed paper-based versions of the same screening tools (ALPHA and EPDS). Both groups completed the screening tools on a single occasion (recruitment).

4b-ii) Report how institutional affiliations are displayed

Report how institutional affiliations are displayed to potential participants [on ehealth media], as affiliations with prestigious hospitals or universities may affect volunteer rates, use, and reactions with regards to an intervention.(Not a required item – describe only if this may bias results)

	1	2	3	4	5	
subitem not at all important	◉	○	○	○	○	essential

Does your paper address subitem 4b-ii?

Copy and paste relevant sections from the manuscript (include quotes in quotation marks "like this" to indicate direct quotes from your manuscript), or elaborate on this item by providing additional information not in the ms, or briefly explain why the item is not applicable/relevant for your study

The University of Alberta logo was present on all copies of consent and information letter.

5) The interventions for each group with sufficient details to allow replication, including how and when they were actually administered

5-i) Mention names, credential, affiliations of the developers, sponsors, and owners

Mention names, credential, affiliations of the developers, sponsors, and owners [6] (if authors/evaluators are owners or developer of the software, this needs to be declared in a “Conflict of interest” section or mentioned elsewhere in the manuscript).

1 2 3 4 5

subitem not at all important (1 selected) 2 3 4 5 essential

Does your paper address subitem 5-i?

Copy and paste relevant sections from the manuscript (include quotes in quotation marks "like this" to indicate direct quotes from your manuscript), or elaborate on this item by providing additional information not in the ms, or briefly explain why the item is not applicable/relevant for your study

The authors used the "redcap" research software managed by the Women's and Children's Health Research Institute team at the University of Alberta.

5-ii) Describe the history/development process

Describe the history/development process of the application and previous formative evaluations (e.g., focus groups, usability testing), as these will have an impact on adoption/use rates and help with interpreting results.

	1	2	3	4	5	
subitem not at all important	◉	○	○	○	○	essential

Does your paper address subitem 5-ii?

Copy and paste relevant sections from the manuscript (include quotes in quotation marks "like this" to indicate direct quotes from your manuscript), or elaborate on this item by providing additional information not in the ms, or briefly explain why the item is not applicable/relevant for your study

REDCap was created in 2004 at Vanderbilt University. It originally supported a small group of clinical researchers who needed a secure data collection tool that met HIPAA compliance standards. REDCap quickly became their go-to method for supporting both single and multi-site research studies.

REDCap's developers firmly believed that nobody could know the research as well as the researcher. So a user-friendly web-based interface was introduced to put the researchers in total control of their work. No background knowledge or technical experience was needed to use REDCap; researchers could directly manage their own projects whenever and however they wished, through any browser on any device.

Vanderbilt was now able to invest minimal institutional resources yet still safely and reliably support an increasing number of research studies in REDCap. They explored ways to disseminate the now mature software, as well as to foster broader collaboration for future development.

5-iii) Revisions and updating

Revisions and updating. Clearly mention the date and/or version number of the application/intervention (and comparator, if applicable) evaluated, or describe whether the intervention underwent major changes during the evaluation process, or whether the development and/or content was “frozen” during the trial.

Describe dynamic components such as news feeds or changing content which may have an impact on the replicability of the intervention (for unexpected events see item 3b).

	1	2	3	4	5	
subitem not at all important	●	○	○	○	○	essential

Does your paper address subitem 5-iii?

Copy and paste relevant sections from the manuscript (include quotes in quotation marks "like this" to indicate direct quotes from your manuscript), or elaborate on this item by providing additional information not in the ms, or briefly explain why the item is not applicable/relevant for your study

The intervention did not undergo any changes during the evaluation process.

5-iv) Quality assurance methods

Provide information on quality assurance methods to ensure accuracy and quality of information provided [1], if applicable.

	1	2	3	4	5	
subitem not at all important	●	○	○	○	○	essential

Does your paper address subitem 5-iv?

Copy and paste relevant sections from the manuscript (include quotes in quotation marks "like this" to indicate direct quotes from your manuscript), or elaborate on this item by providing additional information not in the ms, or briefly explain why the item is not applicable/relevant for your study

Upon submission, survey data were sent to a secure server housed in the Faculty of Medicine and Dentistry at the University of Alberta, through Redcap software.

5-v) Ensure replicability by publishing the source code, and/or providing screenshots/screen-capture video, and/or providing flowcharts of the algorithms used

Ensure replicability by publishing the source code, and/or providing screenshots/screen-capture video, and/or providing flowcharts of the algorithms used. Replicability (i.e., other researchers should in principle be able to replicate the study) is a hallmark of scientific reporting.

	1	2	3	4	5	
subitem not at all important	●	○	○	○	○	essential

Does your paper address subitem 5-v?

Copy and paste relevant sections from the manuscript (include quotes in quotation marks "like this" to indicate direct quotes from your manuscript), or elaborate on this item by providing additional information

not in the ms, or briefly explain why the item is not applicable/relevant for your study

The Redcap software is available throughout the US and Canada.

5-vi) Digital preservation

Digital preservation: Provide the URL of the application, but as the intervention is likely to change or disappear over the course of the years; also make sure the intervention is archived (Internet Archive, webcitation.org, and/or publishing the source code or screenshots/videos alongside the article). As pages behind login screens cannot be archived, consider creating demo pages which are accessible without login.

	1	2	3	4	5	
subitem not at all important	(•)	( )	( )	( )	( )	essential

Does your paper address subitem 5-vi?

Copy and paste relevant sections from the manuscript (include quotes in quotation marks "like this" to indicate direct quotes from your manuscript), or elaborate on this item by providing additional information not in the ms, or briefly explain why the item is not applicable/relevant for your study

Recap url is: https://www.project-redcap.org/

5-vii) Access

Access: Describe how participants accessed the application, in what setting/context, if they had to pay (or were paid) or not, whether they had to be a member of specific group. If known, describe how participants obtained "access to the platform and Internet" [1]. To ensure access for editors/reviewers/readers, consider to provide a "backdoor" login account or demo mode for reviewers/readers to explore the application (also important for archiving purposes, see vi).

	1	2	3	4	5	
subitem not at all important	(•)	( )	( )	( )	( )	essential

Does your paper address subitem 5-vii? *

Copy and paste relevant sections from the manuscript (include quotes in quotation marks "like this" to indicate direct quotes from your manuscript), or elaborate on this item by providing additional information not in the ms, or briefly explain why the item is not applicable/relevant for your study

Redcap is administered through the University of Alberta and WCHRI, Edmonton Alberta although most universities have access to redcap. A personalized link was sent to the intervention participants to directly access the Redcap site. Access to this system is maintained by secure protocols and only accessible by University staff or researchers.

5-viii) Mode of delivery, features/functionalities/components of the intervention and comparator, and the theoretical framework

Describe mode of delivery, features/functionalities/components of the intervention and comparator, and the theoretical framework [6] used to design them (instructional strategy [1], behaviour change techniques, persuasive features, etc., see e.g., [7, 8] for terminology). This includes an in-depth description of the content (including where it is coming from and who developed it) [1],” whether [and how] it is tailored to individual circumstances and allows users to track their progress and receive feedback” [6]. This also includes a description of communication delivery channels and – if computer-mediated communication is a component – whether communication was synchronous or asynchronous [6]. It also includes information on presentation strategies [1], including page design principles, average amount of text on pages, presence of hyperlinks to other resources, etc. [1].

	1	2	3	4	5	
subitem not at all important	(•)	( )	( )	( )	( )	essential

Does your paper address subitem 5-viii? *

Copy and paste relevant sections from the manuscript (include quotes in quotation marks "like this" to indicate direct quotes from your manuscript), or elaborate on this item by providing additional information not in the ms, or briefly explain why the item is not applicable/relevant for your study

The control group received paper versions of the mental health screening and the intervention received an electronic version. Electronic versions were contained on tablets. The functionality of the electronic version was based on Redcap software and any limitations thereof. Individualization of branching and content is managed by University of Alberta staff. For further capabilities of Redcap please see: https://www.project-redcap.org/

5-ix) Describe use parameters

Describe use parameters (e.g., intended “doses” and optimal timing for use). Clarify what instructions or recommendations were given to the user, e.g., regarding timing, frequency, heaviness of use, if any, or was the intervention used ad libitum.

	1	2	3	4	5	
subitem not at all important	( )	(•)	( )	( )	( )	essential

Does your paper address subitem 5-ix?

Copy and paste relevant sections from the manuscript (include quotes in quotation marks "like this" to indicate direct quotes from your manuscript), or elaborate on this item by providing additional information not in the ms, or briefly explain why the item is not applicable/relevant for your study

5-x) Clarify the level of human involvement

Clarify the level of human involvement (care providers or health professionals, also technical assistance) in the e-intervention or as co-intervention (detail number and expertise of professionals involved, if any, as well as “type of assistance offered, the timing and frequency of the support, how it is initiated, and the medium by which the assistance is delivered”. It may be necessary to distinguish between the level of human

involvement required for the trial, and the level of human involvement required for a routine application outside of a RCT setting (discuss under item 21 – generalizability).

	1	2	3	4	5	
subitem not at all important	○	◉	○	○	○	essential

Does your paper address subitem 5-x?

Copy and paste relevant sections from the manuscript (include quotes in quotation marks "like this" to indicate direct quotes from your manuscript), or elaborate on this item by providing additional information not in the ms, or briefly explain why the item is not applicable/relevant for your study

Following consent and computer randomization, women in the intervention group completed the Web-based e-screening version of the ALPHA and EPDS on a computer tablet. They then
proceeded to complete the Web-based baseline questionnaire. Women in the control group completed the Web-based consent on the tablet; thereafter, they were given the paper-based versions of the ALPHA and EPDS. Once finished, they returned to the tablet to complete the Web-based baseline questionnaire. One week after recruitment, women in both groups were telephoned by a trained research assistant (blinded to group allocation) to complete a Mini-International Neuropsychiatric Interview (MINI, Version 6.0.0) [29]. For prenatal classes, the link was sent to the participant via email.

5-xi) Report any prompts/reminders used

Report any prompts/reminders used: Clarify if there were prompts (letters, emails, phone calls, SMS) to use the application, what triggered them, frequency etc. It may be necessary to distinguish between the level of prompts/reminders required for the trial, and the level of prompts/reminders for a routine application outside of a RCT setting (discuss under item 21 – generalizability).

	1	2	3	4	5	
subitem not at all important	◉	○	○	○	○	essential

Does your paper address subitem 5-xi? *

Copy and paste relevant sections from the manuscript (include quotes in quotation marks "like this" to indicate direct quotes from your manuscript), or elaborate on this item by providing additional information not in the ms, or briefly explain why the item is not applicable/relevant for your study

For prenatal class participants, the link was sent to the intervention participants via email and an automated reminder was sent every week.

5-xii) Describe any co-interventions (incl. training/support)

Describe any co-interventions (incl. training/support): Clearly state any interventions that are provided in addition to the targeted eHealth intervention, as ehealth intervention may not be designed as stand-alone intervention. This includes training sessions and support [1]. It may be necessary to distinguish between the

level of training required for the trial, and the level of training for a routine application outside of a RCT setting (discuss under item 21 – generalizability.

	1	2	3	4	5	
subitem not at all important	○	○	●	○	○	essential

Does your paper address subitem 5-xii? *

Copy and paste relevant sections from the manuscript (include quotes in quotation marks "like this" to indicate direct quotes from your manuscript), or elaborate on this item by providing additional information not in the ms, or briefly explain why the item is not applicable/relevant for your study

Training of the RA to conduct a MINI diagnostic interview within 1 week of recruitment.

6a) Completely defined pre-specified primary and secondary outcome measures, including how and when they were assessed

Does your paper address CONSORT subitem 6a? *

Copy and paste relevant sections from the manuscript (include quotes in quotation marks "like this" to indicate direct quotes from your manuscript), or elaborate on this item by providing additional information not in the ms, or briefly explain why the item is not applicable/relevant for your study

We measured women's views of the risk and benefits of e-screening using the 8-item DES. The DES comprises 2 subscales, the risk subscale (items 1, 2, 4, and 5) and the utility
subscale (items 3, 6, 7, and 8), designed to identify the perceived risks and benefits of psychological care. Convergent validity of the subscales has been demonstrated with other measures of self-disclosure, as well as psychological distress and intention to seek mental health care [31]. Instructions preceding the DES asked women to consider each question within the context of discussing mental health problems with their prenatal care provider. The risk subscale assesses the level of risk and consequences women perceive in self-disclosing mental health concerns and is based on the notion that the "potential dangers of opening up to another person may seem to some individuals worse than their actual problem" [31]. The utility subscale measures the perceived value of disclosure. Participants responded to each item on a 5-point Likert scale from "very" to "not at all." The individual scale items are given with their sample distributions in Multimedia Appendix.

6a-i) Online questionnaires: describe if they were validated for online use and apply CHERRIES items to describe how the questionnaires were designed/deployed

If outcomes were obtained through online questionnaires, describe if they were validated for online use and apply CHERRIES items to describe how the questionnaires were designed/deployed [9].

	1	2	3	4	5	
subitem not at all important	○	○	●	○	○	essential

Does your paper address subitem 6a-i?

Copy and paste relevant sections from manuscript text

The surveys were validated for online use by the University of Alberta Research Ethics board.
Participants were informed of the length of time of the survey, where and for how long the data were stored, the investigator and purpose of study by an RA. Data was protected by the University of Alberta Redcap system wtihin the Health data repository in the Faculty of Nursing.
Surveys were developed and tested by Informatics team at the University of Alberta (WCHRI), Edmonton, Alberta.
The survey was a closed survey with initial contact being through a research assistant at maternity clinics, hospital ward and prenatal classes.The tablet delivery system allowed for immediate capture of data to the Redcap system at U of Alberta. No data remained on the tablet. The email sent surveys were a link in which the data was again captured by the Redcap system. This was a volutary survey, with no incentives offered. Data was collected from August 2013 to January 2015.
Participants were randomized but no adaptive questioning was used.
Incomplete questionnaires: All participants who completed at least the first section of the survey (the demographics and randomization) were included in the analysis. All analysis was done using complete case, and incomplete data rates were very low.
We did not timestamp our surveys and there was no weighting of items.
The rest of the CHERRIES checklist we have not yet analyzed the data concerning these items.

6a-ii) Describe whether and how “use” (including intensity of use/dosage) was defined/measured/monitored

Describe whether and how “use” (including intensity of use/dosage) was defined/measured/monitored (logins, logfile analysis, etc.). Use/adoption metrics are important process outcomes that should be reported in any ehealth trial.

	1	2	3	4	5	
subitem not at all important	(●)	( )	( )	( )	( )	essential

Does your paper address subitem 6a-ii?

Copy and paste relevant sections from manuscript text

In terms of risk, the item with the most endorsements was “How vulnerable would you feel if you disclosed something very personal to your doctor or nurse that you have never told anyone before,” with 42.4% (128/302) of women in the e-screening group and 43.8% (144/329) in the paper-based group indicating disclosure of a mental health concern would make them feel somewhat, moderately, or very vulnerable (Multimedia Appendix 1).

6a-iii) Describe whether, how, and when qualitative feedback from participants was obtained

Describe whether, how, and when qualitative feedback from participants was obtained (e.g., through emails, feedback forms, interviews, focus groups).

1 2 3 4 5

subitem not at all important (1) ● ○ ○ ○ ○ essential

Does your paper address subitem 6a-iii?

Copy and paste relevant sections from manuscript text

Qualitative feedback was obtained from participant interviews however, not reported in this article.

6b) Any changes to trial outcomes after the trial commenced, with reasons

Does your paper address CONSORT subitem 6b? *

Copy and paste relevant sections from the manuscript (include quotes in quotation marks "like this" to indicate direct quotes from your manuscript), or elaborate on this item by providing additional information not in the ms, or briefly explain why the item is not applicable/relevant for your study

Not applicable

7a) How sample size was determined

NPT: When applicable, details of whether and how the clustering by care provides or centers was addressed

7a-i) Describe whether and how expected attrition was taken into account when calculating the sample size

Describe whether and how expected attrition was taken into account when calculating the sample size.

	1	2	3	4	5	
subitem not at all important	○	○	○	○	●	essential

Does your paper address subitem 7a-i?

Copy and paste relevant sections from manuscript title (include quotes in quotation marks "like this" to indicate direct quotes from your manuscript), or elaborate on this item by providing additional information not in the ms, or briefly explain why the item is not applicable/relevant for your study

Because no data were available to guide estimation of a minimal clinically important difference in true cases detected through e-screening, we used a CI approach [30]. We based the sample size calculation on 85% of women with a score of 4 to 8 on the risk subscale of the Disclosure Expectations Scale (DES) and 85% of women with a score of 16 to 20 on the utility subscale of the DES. Using a margin of error of 0.05 and 25% estimated loss to follow-up, we calculated that 261 women per group (N=542) were required[23]). At a final sample size of 636, the study was sufficiently powered to detect differences in the outcomes between groups if they exist.

7b) When applicable, explanation of any interim analyses and stopping guidelines

Does your paper address CONSORT subitem 7b? *

Copy and paste relevant sections from the manuscript (include quotes in quotation marks "like this" to indicate direct quotes from your manuscript), or elaborate on this item by providing additional information not in the ms, or briefly explain why the item is not applicable/relevant for your study

No interim analyses or stopping guidelines other than sample size.

8a) Method used to generate the random allocation sequence

NPT: When applicable, how care providers were allocated to each trial group

Does your paper address CONSORT subitem 8a? *

Copy and paste relevant sections from the manuscript (include quotes in quotation marks "like this" to indicate direct quotes from your manuscript), or elaborate on this item by providing additional information not in the ms, or briefly explain why the item is not applicable/relevant for your study

Computer generated radom allocation

8b) Type of randomisation; details of any restriction (such as blocking and block size)

Does your paper address CONSORT subitem 8b? *

Copy and paste relevant sections from the manuscript (include quotes in quotation marks "like this" to indicate direct quotes from your manuscript), or elaborate on this item by providing additional information not in the ms, or briefly explain why the item is not applicable/relevant for your study

Computer generated randomisation group 1 and group 2.

9) Mechanism used to implement the random allocation sequence (such as sequentially numbered containers), describing any steps taken to conceal the sequence until interventions were assigned

Does your paper address CONSORT subitem 9? *

Copy and paste relevant sections from the manuscript (include quotes in quotation marks "like this" to indicate direct quotes from your manuscript), or elaborate on this item by providing additional information not in the ms, or briefly explain why the item is not applicable/relevant for your study

nonapplicable

10) Who generated the random allocation sequence, who enrolled participants, and who assigned participants to interventions

Does your paper address CONSORT subitem 10? *

Copy and paste relevant sections from the manuscript (include quotes in quotation marks "like this" to indicate direct quotes from your manuscript), or elaborate on this item by providing additional information not in the ms, or briefly explain why the item is not applicable/relevant for your study

The redcap system generated the random allocation sequence when the participant hit on the link. This automatically entrolled the participants and randomized the participants immediately at the beginning of the survey.

11a) If done, who was blinded after assignment to interventions (for example, participants, care providers, those assessing outcomes) and how

NPT: Whether or not administering co-interventions were blinded to group assignment

11a-i) Specify who was blinded, and who wasn't

Specify who was blinded, and who wasn't. Usually, in web-based trials it is not possible to blind the participants [1, 3] (this should be clearly acknowledged), but it may be possible to blind outcome assessors, those doing data analysis or those administering co-interventions (if any).

	1	2	3	4	5	
subitem not at all important	○	○	●	○	○	essential

Does your paper address subitem 11a-i? *

Copy and paste relevant sections from the manuscript (include quotes in quotation marks "like this" to indicate direct quotes from your manuscript), or elaborate on this item by providing additional information not in the ms, or briefly explain why the item is not applicable/relevant for your study

The RA, care providers were blinded, but the participants were not, obviously, as it was an RCT.

11a-ii) Discuss e.g., whether participants knew which intervention was the "intervention of interest" and which one was the "comparator"

Informed consent procedures (4a-ii) can create biases and certain expectations - discuss e.g., whether participants knew which intervention was the "intervention of interest" and which one was the "comparator".

	1	2	3	4	5	
subitem not at all important	○	○	●	○	○	essential

Does your paper address subitem 11a-ii?

Copy and paste relevant sections from the manuscript (include quotes in quotation marks "like this" to indicate direct quotes from your manuscript), or elaborate on this item by providing additional information not in the ms, or briefly explain why the item is not applicable/relevant for your study

The participants did not know which intervention was the intervention of interest except that the tablet was a newer form of delivery that they had not seen before in a research context.

11b) If relevant, description of the similarity of interventions

(this item is usually not relevant for ehealth trials as it refers to similarity of a placebo or sham intervention to a active medication/intervention)

Does your paper address CONSORT subitem 11b? *

Copy and paste relevant sections from the manuscript (include quotes in quotation marks "like this" to indicate direct quotes from your manuscript), or elaborate on this item by providing additional information not in the ms, or briefly explain why the item is not applicable/relevant for your study

Not applicable.

12a) Statistical methods used to compare groups for primary and secondary outcomes

NPT: When applicable, details of whether and how the clustering by care providers or centers was addressed

Does your paper address CONSORT subitem 12a? *

Copy and paste relevant sections from the manuscript (include quotes in quotation marks "like this" to indicate direct quotes from your manuscript), or elaborate on this item by providing additional information not in the ms, or briefly explain why the item is not applicable/relevant for your study

Because there was a little data missing, we conducted a completer analysis (vs intention-to-treat analysis). Baseline differences of the groups were assessed using frequencies (95% CIs) and means (standard deviations [SD]) and compared using independent t tests (means) and chi-square tests (%) to assess the effectiveness of randomization. Statistical significance for

all analyses and final models was set at P<.05. We used chi-square tests to compare proportions of women in each group responding to the subscale items.
Before the multivariable analysis, we conducted bivariate analyses to identify independent factors that were significantly associated with each of the outcomes at P<.20, estimating unadjusted odds ratios and their 95% CIs. Those variables were entered in the final multivariable models simultaneously, where P<.05 defined factors that were significantly associated with the outcomes in the final models.
Results

Sample Characteristics
Of the 675 eligible women approached from August 2013 to January 2015, 636 agreed to participate (participation rate: 94.2%, 636/675) and were randomized to the intervention (n=305) and control (n=331) groups. A total of 5 women withdrew from the study following group allocation: 3 in the intervention group and 2 in the control group (see Figure 1). There were no statistically significant differences at baseline between the two groups. Table 1 shows that the majority of pregnant women were between 25 and 34 years of age, partnered, white, had incomes of Can $80,000 or more, had at least some postsecondary education and were pregnant with their first child. One-quarter of participants had been diagnosed and treated for a mental health concern before recruitment. The majority of women were comfortable using laptops, computer tablets, and smartphones. Missing data were less than 3.0% (19/636) for all variables, with the majority having less than 1.5% (10/636); thus, data imputation was not used.

Primary and Secondary Objectives
Primary Objectives
Perceived Risk and Benefit of Disclosure: Description of Items of the Risk and Utility Subscales
There were no significant differences between groups on any of the items of the risk or benefit subscales of the DES (Multimedia Appendix 1). In terms of risk, the item with the most endorsements was "How vulnerable would you feel if you disclosed something very personal to your doctor or nurse that you have never told anyone before," with 42.4% (128/302) of women in the e-screening group and 43.8% (144/329) in the paper-based group indicating disclosure of a mental health concern would make them feel somewhat, moderately, or very vulnerable (Multimedia Appendix 1). This was followed by women endorsing that they would perceive disclosure as somewhat or moderately or very "risky" (e-screening 34.4% [104/302]; paper 35.3% [116/329]), "worrisome" (e-screening 29.5% [89/302]; paper 32.5% [107/329]), and "difficult" (e-screening 22.2% [67/302]; paper 21.0% [69/329])
(Multimedia Appendix 1).
From a benefits perspective, the majority of women in both groups felt they would get a useful response from their provider if they disclosed their concerns (e-screening 81.1% [245/302];

paper 83.9% [276/329]), and it would be beneficial to do so (e-screening 83.1% [251/302]; paper 81.5% [268/329]). Additionally, 76.8% (485/631) of women felt that it would be helpful to talk to their provider about a mental health problem

(e-screening 76.2% [230/302]; paper 77.5% [255/329]), and it would feel better to have the opportunity to discuss their feelings of anxiety or depression with them (e-screening 70.9% [214/302]; paper 77.5% [255/329]).

12a-i) Imputation techniques to deal with attrition / missing values

Imputation techniques to deal with attrition / missing values: Not all participants will use the intervention/comparator as intended and attrition is typically high in ehealth trials. Specify how participants who did not use the application or dropped out from the trial were treated in the statistical analysis (a complete case analysis is strongly discouraged, and simple imputation techniques such as LOCF may also be problematic [4]).

	1	2	3	4	5	
subitem not at all important	○	○	○	○	○	essential

Does your paper address subitem 12a-i? *

Copy and paste relevant sections from the manuscript (include quotes in quotation marks "like this" to indicate direct quotes from your manuscript), or elaborate on this item by providing additional information not in the ms, or briefly explain why the item is not applicable/relevant for your study

Because there was a little data missing, we conducted a completer analysis (vs intention-to-treat analysis). Baseline differences of the groups were assessed using frequencies (95% CIs) and means (standard deviations [SD]) and compared using independent t tests (means) and chi-square tests (%) to assess the effectiveness of randomization. Statistical significance for

all analyses and final models was set at P<.05. We used chi-square tests to compare proportions of women in each group responding to the subscale items.
Before the multivariable analysis, we conducted bivariate analyses to identify independent factors that were significantly associated with each of the outcomes at P<.20, estimating unadjusted odds ratios and their 95% CIs. Those variables were entered in the final multivariable models simultaneously, where P<.05 defined factors that were significantly associated with the outcomes in the final models.
Results

Sample Characteristics
Of the 675 eligible women approached from August 2013 to January 2015, 636 agreed to participate (participation rate: 94.2%, 636/675) and were randomized to the intervention (n=305) and control (n=331) groups. A total of 5 women withdrew from the study following group allocation: 3 in the intervention group and 2 in the control group (see Figure 1). There were no statistically significant differences at baseline between the two groups.
Table 1 shows that the majority of pregnant women were between 25 and 34 years of age, partnered, white, had incomes of Can $80,000 or more, had at least some postsecondary education and were pregnant with their first child. One-quarter of participants had been diagnosed and treated for a mental health concern before recruitment. The majority of women were comfortable using laptops, computer tablets, and smartphones. Missing data were less than 3.0% (19/636) for all variables, with the majority having less than 1.5% (10/636); thus, data imputation was not used.
Primary and Secondary Objectives
Primary Objectives
Perceived Risk and Benefit of Disclosure: Description of Items of the Risk and Utility Subscales
There were no significant differences between groups on any of the items of the risk or benefit subscales of the DES (Multimedia Appendix 1). In terms of risk, the item with the most endorsements was “How vulnerable would you feel if you disclosed something very personal to your doctor or nurse that you have never told anyone before,” with 42.4% (128/302) of women in the e-screening group and 43.8% (144/329) in the paper-based group indicating disclosure of a mental health concern would make them feel somewhat, moderately, or very vulnerable (Multimedia Appendix 1). This was followed by women endorsing that they would perceive disclosure as somewhat or moderately or very “risky” (e-screening 34.4% [104/302]; paper 35.3% [116/329]), “worrisome” (e-screening 29.5% [89/302]; paper 32.5% [107/329]), and “difficult” (e-screening 22.2% [67/302]; paper 21.0% [69/329])
(Multimedia Appendix 1).
From a benefits perspective, the majority of women in both groups felt they would get a useful response from their provider if they disclosed their concerns (e-screening 81.1% [245/302];

paper 83.9% [276/329], and it would be beneficial to do so (e-screening

12b) Methods for additional analyses, such as subgroup analyses and adjusted analyses

Does your paper address CONSORT subitem 12b? *

Copy and paste relevant sections from the manuscript (include quotes in quotation marks "like this" to indicate direct quotes from your manuscript), or elaborate on this item by providing additional information not in the ms, or briefly explain why the item is not applicable/relevant for your study

not applicable

X26) REB/IRB Approval and Ethical Considerations [recommended as subheading under "Methods"] (not a CONSORT item)

X26-i) Comment on ethics committee approval

	1	2	3	4	5	
subitem not at all important	○	○	◉	○	○	essential

Does your paper address subitem X26-i?

Copy and paste relevant sections from the manuscript (include quotes in quotation marks "like this" to indicate direct quotes from your manuscript), or elaborate on this item by providing additional information not in the ms, or briefly explain why the item is not applicable/relevant for your study

Obtaining Ethics for electronic consent was approved by Research Ethics board at the university of Alberta.

x26-ii) Outline informed consent procedures

Outline informed consent procedures e.g., if consent was obtained offline or online (how? Checkbox, etc.?), and what information was provided (see 4a-ii). See [6] for some items to be included in informed consent documents.

	1	2	3	4	5	
subitem not at all important	○	○	○	◉	○	essential

Does your paper address subitem X26-ii?

Copy and paste relevant sections from the manuscript (include quotes in quotation marks "like this" to indicate direct quotes from your manuscript), or elaborate on this item by providing additional information not in the ms, or briefly explain why the item is not applicable/relevant for your study

Consent was obtained online for the tablet use and offline with paper survey.

X26-iii) Safety and security procedures

Safety and security procedures, incl. privacy considerations, and any steps taken to reduce the likelihood or detection of harm (e.g., education and training, availability of a hotline)

	1	2	3	4	5	
subitem not at all important	○	○	●	○	○	essential

Does your paper address subitem X26-iii?

Copy and paste relevant sections from the manuscript (include quotes in quotation marks "like this" to indicate direct quotes from your manuscript), or elaborate on this item by providing additional information not in the ms, or briefly explain why the item is not applicable/relevant for your study

Privacy was maintained through the Redcap system.
Safety Protocol
Women who met criteria for a mood or anxiety disorder on the MINI or scored 13 or more on the EPDS were referred by the research assistant to the hospital-based reproductive mental health.

RESULTS

13a) For each group, the numbers of participants who were randomly assigned, received intended treatment, and were analysed for the primary outcome

NPT: The number of care providers or centers performing the intervention in each group and the number of patients treated by each care provider in each center

Does your paper address CONSORT subitem 13a? *

Copy and paste relevant sections from the manuscript (include quotes in quotation marks "like this" to indicate direct quotes from your manuscript), or elaborate on this item by providing additional information not in the ms, or briefly explain why the item is not applicable/relevant for your study

Of the 675 eligible women approached from August 2013 to January 2015, 636 agreed to participate (participation rate: 94.2%, 636/675) and were randomized to the intervention (n=305) and control (n=331) groups. A total of 5 women withdrew from the study following group allocation: 3 in the intervention group and 2 in the control group (see Figure 1). There were no statistically significant differences at baseline between the two groups.

13b) For each group, losses and exclusions after randomisation, together with reasons

Does your paper address CONSORT subitem 13b? (NOTE: Preferably, this is shown in a CONSORT flow diagram) *

Copy and paste relevant sections from the manuscript (include quotes in quotation marks "like this" to indicate direct quotes from your manuscript), or elaborate on this item by providing additional information not in the ms, or briefly explain why the item is not applicable/relevant for your study

Of the 675 eligible women approached from August 2013 to January 2015, 636 agreed to participate (participation rate: 94.2%, 636/675) and were randomized to the intervention (n=305) and control (n=331) groups. A total of 5 women withdrew from the study following group allocation: 3 in the intervention group and 2 in the control group (see Figure 1). There were no statistically significant differences at baseline between the two groups.

13b-i) Attrition diagram

Strongly recommended: An attrition diagram (e.g., proportion of participants still logging in or using the intervention/comparator in each group plotted over time, similar to a survival curve) or other figures or tables demonstrating usage/dose/engagement.

	1	2	3	4	5	
subitem not at all important	(•)	( )	( )	( )	( )	essential

Does your paper address subitem 13b-i?

Copy and paste relevant sections from the manuscript or cite the figure number if applicable (include quotes in quotation marks "like this" to indicate direct quotes from your manuscript), or elaborate on this item by providing additional information not in the ms, or briefly explain why the item is not applicable/relevant for your study

nonapplicable

14a) Dates defining the periods of recruitment and follow-up

Does your paper address CONSORT subitem 14a? *

Copy and paste relevant sections from the manuscript (include quotes in quotation marks "like this" to indicate direct quotes from your manuscript), or elaborate on this item by providing additional information not in the ms, or briefly explain why the item is not applicable/relevant for your study

August 2013 to January 2015,

14a-i) Indicate if critical “secular events” fell into the study period

Indicate if critical “secular events” fell into the study period, e.g., significant changes in Internet resources available or “changes in computer hardware or Internet delivery resources”

	1	2	3	4	5	
subitem not at all important	(•)	( )	( )	( )	( )	essential

Does your paper address subitem 14a-i?

Copy and paste relevant sections from the manuscript (include quotes in quotation marks "like this" to indicate direct quotes from your manuscript), or elaborate on this item by providing additional information not in the ms, or briefly explain why the item is not applicable/relevant for your study

nonapplicable

14b) Why the trial ended or was stopped (early)

Does your paper address CONSORT subitem 14b? *

Copy and paste relevant sections from the manuscript (include quotes in quotation marks "like this" to indicate direct quotes from your manuscript), or elaborate on this item by providing additional information not in the ms, or briefly explain why the item is not applicable/relevant for your study

non-applicable. Trial was ended when we reached our sample size.

15) A table showing baseline demographic and clinical characteristics for each group

NPT: When applicable, a description of care providers (case volume, qualification, expertise, etc.) and centers (volume) in each group

Does your paper address CONSORT subitem 15? *

Copy and paste relevant sections from the manuscript (include quotes in quotation marks "like this" to indicate direct quotes from your manuscript), or elaborate on this item by providing additional information not in the ms, or briefly explain why the item is not applicable/relevant for your study

Table 1. Sample characteristics (N=636).
E-screening group P valueb
(n=305a)
Paper-based screening group
(n=331a)
Full sample
(N=636a)
Characteristics
Recruitment site, n (%)
Community-based clinic 423 (67.8) 224 (70.0) 199 (65.5) .47
High-risk antenatal unit 70 (11.2) 34 (10.6) 36 (11.8)
Prenatal class, n (%) 131 (21.0) 62 (19.4) 69 (22.7)
Age, n (%)
<25 years 88 (13.9) 50 (15.2) 38 (12.5) .51
25-34 years 459 (72.2) 233 (70.6) 226 (74.6)
35+ 86 (13.6) 47 (14.2) 39 (12.9)
Income, n (%)
Below $40,000 97 (15.4) 52 (15.8) 45 (14.9) .81
$40,000-$79,999 139 (22.0) 75 (22.8) 64 (21.2)
$80,000 or more 395 (62.6) 202 (61.4) 193 (63.9)
Education, n (%)
High school or less 100 (15.8) 57 (17.3) 43 (14.2) .29
Some postsecondary or more 531 (84.2) 272 (82.7) 259 (85.8)
Marital status, n (%)
Unpartnered 27 (4.3) 14 (4.3) 13 (4.3) .98
Partnered 604 (95.7) 315 (95.7) 289 (95.7)
Ethnicity, n (%)
Not white 169 (26.8) 91 (27.7) 78 (25.8) .60
white 462 (73.2) 238 (72.3) 224 (74.2)
Born in Canada, n (%)
No 119 (18.9) 66 (20.1) 53 (17.5) .42
Yes 512 (81.1) 263 (79.9) 249 (82.5)
Ever diagnosed with depression, anxiety, or any other kind
of emotional concern, n (%)
Yes 164 (25.9) 86 (26.1) 78 (25.7) .91
No 470 (74.1) 244 (73.9) 226 (74.3)
Ever treated for depression, anxiety, or any other kind of
emotional concern, n (%)
Yes 179 (28.2) 92 (27.9) 87 (28.6) .84
No 455 (71.8) 238 (72.1) 217 (71.4)
Pregnant before, n (%)
First child 426 (69.3) 213 (68.5) 213 (70.1) .67
Not first child 189 (30.7) 98 (31.5) 91 (29.9)
Weeks gestation, mean (SDc) 9.00 (6.46) 8.61 (6.08) 9.39 (6.80) .22
Used fertility treatments to become pregnant, n (%)
Yes 35 (5.5) 17 (5.2) 18 (5.9) .67
No 599 (94.5) 313 (94.8) 286 (94.1)
ACEs d score n (%)
Score greater than or equal to 4 113 (18.0) 64 (19.5) 49 (16.3) .31
http://mental.jmir.org/2017/4/e42/ JMIR Ment Health 2017 | vol. 4 | iss. 4 |
e42 | p.6
(page number not for citation purposes)
JMIR MENTAL HEALTH Kingston et al
XSL•FO
RenderX
E-screening group P valueb
(n=305a)
Paper-based screening group
(n=331a)
Full sample
(N=636a)

(N=630a)
Characteristics
Score less than 4 516 (82.0) 265 (80.5) 251 (83.7)
I am comfortable using a computer or laptop, n (%)
Very comfortable 591 (93.7) 311 (94.5) 280 (92.7) .45
Somewhat comfortable 36 (5.7) 17 (5.2) 19 (6.3)
Not very comfortable 4 (0.6) 1 (0.3) 3 (1.0)
I am comfortable using a computer tablet (eg, iPad), n (%)
Very comfortable 530 (84.0) 280 (85.1) 250 (82.8) .64
Somewhat comfortable 89 (14.1) 44 (13.4) 45 (14.9)
Not very comfortable 12 (1.9) 5 (1.5) 7 (2.3)
I am comfortable using a mobile phone, n (%)
Very comfortable 546 (86.5) 286 (86.9) 260 (86.1) .32
Somewhat comfortable 70 (11.1) 38 (11.6) 32 (10.6)
Not very comfortable 15 (2.4) 5 (1.5) 10 (3.3)
aSome demographic data missing.
bComparison of control and intervention groups: χ2 statistic used for variables with three or more categories; two-tailed t test used for variables with
estimated means.
cSD: standard deviation.
dACEs: adverse childhood experiences.

15-i) Report demographics associated with digital divide issues

In ehealth trials it is particularly important to report demographics associated with digital divide issues, such as age, education, gender, social-economic status, computer/Internet/ehealth literacy of the participants, if known.

	1	2	3	4	5	
subitem not at all important	○	○	○	○	○	essential

Does your paper address subitem 15-i? *

Copy and paste relevant sections from the manuscript (include quotes in quotation marks "like this" to indicate direct quotes from your manuscript), or elaborate on this item by providing additional information not in the ms, or briefly explain why the item is not applicable/relevant for your study

We did not ask about computer/internet/ ehealth literacy of participants, just access to computer/device/internet.

16) For each group, number of participants (denominator) included in each analysis and whether the analysis was by original assigned groups

16-i) Report multiple "denominators" and provide definitions

Report multiple "denominators" and provide definitions: Report N's (and effect sizes) "across a range of study participation [and use] thresholds" [1], e.g., N exposed, N consented, N used more than x times, N used more than y weeks, N participants "used" the intervention/comparator at specific pre-defined time

points of interest (in absolute and relative numbers per group). Always clearly define “use” of the intervention.

	1	2	3	4	5	
subitem not at all important	◉	○	○	○	○	essential

Does your paper address subitem 16-i? *

Copy and paste relevant sections from the manuscript (include quotes in quotation marks "like this" to indicate direct quotes from your manuscript), or elaborate on this item by providing additional information not in the ms, or briefly explain why the item is not applicable/relevant for your study

Of the 675 eligible women approached from August 2013 to January 2015, 636 agreed to participate (participation rate: 94.2%, 636/675) and were randomized to the intervention (n=305) and control (n=331) groups

16-ii) Primary analysis should be intent-to-treat

Primary analysis should be intent-to-treat, secondary analyses could include comparing only “users”, with the appropriate caveats that this is no longer a randomized sample (see 18-i).

	1	2	3	4	5	
subitem not at all important	○	○	◉	○	○	essential

Does your paper address subitem 16-ii?

Copy and paste relevant sections from the manuscript (include quotes in quotation marks "like this" to indicate direct quotes from your manuscript), or elaborate on this item by providing additional information not in the ms, or briefly explain why the item is not applicable/relevant for your study

Because there was a little data missing, we conducted a completer analysis (vs intention-to-treat analysis). Baseline differences of the groups were assessed using frequencies (95% CIs) and means (standard deviations [SD]) and compared using independent t tests (means) and chi-square tests (%) to assess the effectiveness of randomization. Statistical significance for

all analyses and final models was set at P<.05. We used chi-square tests to compare proportions of women in each group responding to the subscale items.
Before the multivariable analysis, we conducted bivariate analyses to identify independent factors that were significantly associated with each of the outcomes at P<.20, estimating unadjusted odds ratios and their 95% CIs. Those variables were entered in the final multivariable models simultaneously, where P<.05 defined factors that were significantly associated with the outcomes in the final models.

Primary and Secondary Objectives
Primary Objectives
Perceived Risk and Benefit of Disclosure: Description of Items of the Risk and Utility Subscales
There were no significant differences between groups on any of the items of the risk or benefit subscales of the DES (Multimedia Appendix 1). In terms of risk, the item with the most endorsements was “How vulnerable would you feel if you disclosed something very personal to your doctor or nurse that you have never told anyone before,” with 42.4% (128/302) of women in the e-screening group and 43.8% (144/329) in the paper-based group indicating disclosure of a mental health concern would make them feel somewhat, moderately, or very vulnerable (Multimedia Appendix 1). This was followed by women endorsing that they would perceive disclosure as somewhat or moderately or very “risky” (e-screening 34.4% [104/302]; paper 35.3% [116/329]), “worrisome” (e-screening 29.5% [89/302]; paper 32.5% [107/329]), and “difficult” (e-screening 22.2% [67/302]; paper 21.0% [69/329])
(Multimedia Appendix 1).
From a benefits perspective, the majority of women in both groups felt they would get a useful response from their provider if they disclosed their concerns (e-screening 81.1% [245/302];

paper 83.9% [276/329]), and it would be beneficial to do so (e-screening 83.1% [251/302]; paper 81.5% [268/329]). Additionally, 76.8% (485/631) of women felt that it would be helpful to talk to their provider about a mental health problem

(e-screening 76.2% [230/302]; paper 77.5% [255/329]), and it would feel better to have the opportunity to discuss their feelings of anxiety or depression with them (e-screening 70.9% [214/302]; paper 77.5% [255/329]).

17a) For each primary and secondary outcome, results for each group, and the estimated effect size and its precision (such as 95% confidence interval)

Does your paper address CONSORT subitem 17a? *

Copy and paste relevant sections from the manuscript (include quotes in quotation marks "like this" to indicate direct quotes from your manuscript), or elaborate on this item by providing additional information not in the ms, or briefly explain why the item is not applicable/relevant for your study

Primary and Secondary Objectives
Primary Objectives
Perceived Risk and Benefit of Disclosure: Description of Items of the Risk and Utility Subscales
There were no significant differences between groups on any of the items of the risk or benefit subscales of the DES (Multimedia Appendix 1). In terms of risk, the item with the most endorsements was “How vulnerable would you feel if you disclosed something very personal to your doctor or nurse that you have never told anyone before,” with 42.4% (128/302) of women in the e-screening group and 43.8% (144/329) in the paper-based group indicating disclosure of a mental health concern would make them feel somewhat, moderately, or very vulnerable (Multimedia Appendix 1). This was followed by women endorsing that they would perceive disclosure as somewhat or moderately or very “risky” (e-screening 34.4% [104/302]; paper 35.3% [116/329]), “worrisome” (e-screening 29.5% [89/302]; paper 32.5% [107/329]), and “difficult” (e-screening 22.2% [67/302]; paper 21.0% [69/329])
(Multimedia Appendix 1).
From a benefits perspective, the majority of women in both groups felt they would get a useful response from their provider if they disclosed their concerns (e-screening 81.1% [245/302];

paper 83.9% [276/329]), and it would be beneficial to do so (e-screening 83.1% [251/302]; paper 81.5% [268/329]). Additionally, 76.8% (485/631) of women felt that it would be helpful to talk to their provider about a mental health problem

(e-screening 76.2% [230/302]; paper 77.5% [255/329]), and it would feel better to have the opportunity to discuss their feelings of anxiety or depression with them (e-screening 70.9% [214/302]; paper 77.5% [255/329]).

17a-i) Presentation of process outcomes such as metrics of use and intensity of use

In addition to primary/secondary (clinical) outcomes, the presentation of process outcomes such as metrics of use and intensity of use (dose, exposure) and their operational definitions is critical. This does not only refer to metrics of attrition (13-b) (often a binary variable), but also to more continuous exposure metrics such as “average session length”. These must be accompanied by a technical description how a metric like a “session” is defined (e.g., timeout after idle time) [1] (report under item 6a).

	1	2	3	4	5	
subitem not at all important	○	○	○	○	○	essential

Does your paper address subitem 17a-i?

Copy and paste relevant sections from the manuscript (include quotes in quotation marks "like this" to indicate direct quotes from your manuscript), or elaborate on this item by providing additional information not in the ms, or briefly explain why the item is not applicable/relevant for your study

We did not study the metrics of use or intensity of use. Participants completed one survey and did not have repeat usage.

17b) For binary outcomes, presentation of both absolute and relative effect sizes is recommended

Does your paper address CONSORT subitem 17b? *

Copy and paste relevant sections from the manuscript (include quotes in quotation marks "like this" to indicate direct quotes from your manuscript), or elaborate on this item by providing additional information not in the ms, or briefly explain why the item is not applicable/relevant for your study

Not applicable

18) Results of any other analyses performed, including subgroup analyses and adjusted analyses, distinguishing pre-specified from exploratory

Does your paper address CONSORT subitem 18? *

Copy and paste relevant sections from the manuscript (include quotes in quotation marks "like this" to indicate direct quotes from your manuscript), or elaborate on this item by providing additional information not in the ms, or briefly explain why the item is not applicable/relevant for your study

Non-applicable

18-i) Subgroup analysis of comparing only users

A subgroup analysis of comparing only users is not uncommon in ehealth trials, but if done, it must be stressed that this is a self-selected sample and no longer an unbiased sample from a randomized trial (see 16-iii).

	1	2	3	4	5	
subitem not at all important	(●)	( )	( )	( )	( )	essential

Does your paper address subitem 18-i?

Copy and paste relevant sections from the manuscript (include quotes in quotation marks "like this" to indicate direct quotes from your manuscript), or elaborate on this item by providing additional information not in the ms, or briefly explain why the item is not applicable/relevant for your study

Not applicable

19) All important harms or unintended effects in each group

(for specific guidance see CONSORT for harms)

Does your paper address CONSORT subitem 19? *

Copy and paste relevant sections from the manuscript (include quotes in quotation marks "like this" to indicate direct quotes from your manuscript), or elaborate on this item by providing additional information not in the ms, or briefly explain why the item is not applicable/relevant for your study

Our safety protocol mitigated any real or imagined harms.
Women who met criteria for a mood or anxiety disorder on the MINI or scored 13 or more on the EPDS were referred by the research assistant to the hospital-based reproductive mental health.

19-i) Include privacy breaches, technical problems

Include privacy breaches, technical problems. This does not only include physical "harm" to participants, but also incidents such as perceived or real privacy breaches [1], technical problems, and other unexpected/unintended incidents. "Unintended effects" also includes unintended positive effects [2].

	1	2	3	4	5	
subitem not at all important	○	○	◉	○	○	essential

Does your paper address subitem 19-i?

Copy and paste relevant sections from the manuscript (include quotes in quotation marks "like this" to indicate direct quotes from your manuscript), or elaborate on this item by providing additional information not in the ms, or briefly explain why the item is not applicable/relevant for your study

NO privacy breaches

19-ii) Include qualitative feedback from participants or observations from staff/researchers

Include qualitative feedback from participants or observations from staff/researchers, if available, on strengths and shortcomings of the application, especially if they point to unintended/unexpected effects or uses. This includes (if available) reasons for why people did or did not use the application as intended by the developers.

	1	2	3	4	5	
subitem not at all important	(•)	( )	( )	( )	( )	essential

Does your paper address subitem 19-ii?

Copy and paste relevant sections from the manuscript (include quotes in quotation marks "like this" to indicate direct quotes from your manuscript), or elaborate on this item by providing additional information not in the ms, or briefly explain why the item is not applicable/relevant for your study

Qualitative paper will follow.

DISCUSSION

22) Interpretation consistent with results, balancing benefits and harms, and considering other relevant evidence

NPT: In addition, take into account the choice of the comparator, lack of or partial blinding, and unequal expertise of care providers or centers in each group

22-i) Restate study questions and summarize the answers suggested by the data, starting with primary outcomes and process outcomes (use)

Restate study questions and summarize the answers suggested by the data, starting with primary outcomes and process outcomes (use).

	1	2	3	4	5	
subitem not at all important	( )	( )	(•)	( )	( )	essential

Does your paper address subitem 22-i? *

Copy and paste relevant sections from the manuscript (include quotes in quotation marks "like this" to indicate direct quotes from your manuscript), or elaborate on this item by providing additional information not in the ms, or briefly explain why the item is not applicable/relevant for your study

This trial adds substantially to the limited evidence on implementation of screening during the perinatal period by providing data on women's views of the benefits and risks of disclosure of mental health concerns by mode of screening. In this study, 76.8 (485/631) of women perceived that mental health screening was beneficial. However, 21.6% (136/631) to 43.1% (272/631) of women perceived that disclosure held some degree of risk in that they viewed it as risky and worrisome, reporting that it made them feel vulnerable. There were no differences in groups in the mean scores of the risk and benefit of disclosure subscales. In multivariable linear regression analyses, we found that women of low income, those who had been treated [illegible]

analyses, we found that women of low income, those who had been treated previously for depression or anxiety, and those pregnant with their first child were more likely to perceive a greater risk in disclosing mental health concerns compared with women of higher income, who had never been treated for mental health problems, and who were multiparous. We found no factors that were associated with perceiving benefit in screening. Mode of screening (paper-based vs e-screening) was not significantly associated with either perceived risk or benefit of screening.

Overall, pregnant women perceived both paper-based and e-mental health screening to be beneficial. These findings are consistent with our cross-sectional study (N=460), where 97.6% (449/460) of pregnant women surveyed reported that they were very or somewhat comfortable with completing paper-based screening at home (92.3%, 425/460) or in a maternity clinic (90.4%, 416/460), as well as computer-based (86.0%, 395/460) screening [33]. They are also consistent with the study's finding that 97.3% (448/460) of pregnant women were comfortable

with provider-initiated screening, whereas only two-thirds were comfortable with self-initiating discussions about their mental health concerns. Others have also reported a general acceptability of routine mental health screening in Australia, following the initiation of universal prenatal screening through the National Depression Initiative [34-37] and in the United States in hospital-based [14] and regional perinatal screening programs [38].

Women's views of the benefits of screening did not vary by mode of screening. This result indicates that the way women were screened (paper or e-screening) did not influence the value of screening that women perceived in terms of its overall benefit, usefulness, helpfulness, or contribution in making them feel better. This positive finding suggests that whatever mode of screening providers choose to implement in their clinical settings will be viewed as beneficial by women. Similarly, the nonsignificant difference in the mean scores of the risk subscale reveals that women in the paper-based and e-screening groups viewed the degree of risk of disclosure similarly. On one hand, this is positive in that the providers can be assured that the risk that women perceive is independent of the mode of screening they choose to employ in their clinical settings.

However, it is concerning that 43.1% (272/631) of women find screening a vulnerable process. Again, that a similar number of women in both groups reported some degree of vulnerability indicates that this was unrelated to the way the screening questions were delivered and more likely linked to other aspects of the screening process such as the way screening is introduced or debriefed, provider characteristics, or the provider-client relationship. Several studies have shown the importance of provider characteristics and relationships on screening, including being heard and trusting the provider [39], the ability of the

provider to make a connection, being empathetic [40] and being a "good fit" (eg, we "clicked") [13] were key aspects of successful treatment, whereas friendly, sensitive, warm, and caring attributes facilitated the screening process [41]. Conversely, negative experiences with perinatal health care providers have also been shown as detrimental to addressing perinatal depression, including women having their concerns dismissed, perceiving that their provider was inadequately prepared to assess and discuss perinatal depression, being unprepared for the process or the nature of the questions, feeling anxious and vulnerable when raising distressing histories, and seeing the screening process as intrusive [42]. Our own studies mirror these findings. We reported that women who had a relationship with their provider that fostered honesty were less likely to be deterred by potential barriers to screening [15,33,43], and those who had a sensitive and caring and interested provider were more likely to engage in screening [15,33,43]. These studies all support the conclusion that "the way in which clinicians interact with patients about depression might strongly influence patient responses" [39]. Our research has also shown that women were more likely to engage in screening if certain aspects of

[illegible]
the process were in place, such as having an explanation about why some sensitive questions were asked, knowing what to expect if she revealed emotional struggles, being reassured that other women also have prenatal emotional problems, and knowing that talking about emotional health is a part of routine prenatal care [15].
We might have seen a difference in vulnerability by screening mode if we had included a face-to-face screening arm. For instance, qualitative studies of postpartum women have reported that face-to-face screening and discussions around treatment make women feel significantly vulnerable [44,45]. The findings of this study support the importance of the screening process as a whole, in that the mode of screening alone (e-screening vs paper) does not seem to mitigate the vulnerability that women experience during mental health screening.

Although the effect sizes were small, the findings that women of low income, those who had been treated previously for depression or anxiety, and those pregnant with their first child were more likely to perceive a greater risk in disclosing mental health concerns are important in identifying potential subgroups of women who may find screening a more vulnerable process. Given that our sample was quite demographically homogeneous, further research on the views of screening among these subgroups of women is warranted.
Of importance, this study demonstrated that mode of screening was not associated with perceived risk of screening. This finding is positive in light of how little we know about how women perceive e-screening and suggests that e-screening is a viable option for delivering mental health screening. Finally, that no subgroups of women were identified as perceiving greater or less benefit from screening suggests that all women, regardless of demographics or previous mental health history, find mental health screening beneficial. Mode of screening was also not identified as having an impact on perceived benefit, indicating that women find equal benefit from screening regardless of whether the questions are delivered on paper or tablet.

22-ii) Highlight unanswered new questions, suggest future research

Highlight unanswered new questions, suggest future research.

	1	2	3	4	5	
subitem not at all important	○	○	○	○	◉	essential

Does your paper address subitem 22-ii?

Copy and paste relevant sections from the manuscript (include quotes in quotation marks "like this" to indicate direct quotes from your manuscript), or elaborate on this item by providing additional information not in the ms, or briefly explain why the item is not applicable/relevant for your study

Use of e-screening can be an acceptable, resource-sparing option for caring for pregnant women's mental health in primary care settings.

20) Trial limitations, addressing sources of potential bias, imprecision, and, if relevant, multiplicity of analyses

20-i) Typical limitations in ehealth trials

Typical limitations in ehealth trials: Participants in ehealth trials are rarely blinded. Ehealth trials often look at a multiplicity of outcomes, increasing risk for a Type I error. Discuss biases due to non-use of the intervention/usability issues, biases through informed consent procedures, unexpected events.

	1	2	3	4	5	
subitem not at all important	(•)	( )	( )	( )	( )	essential

Does your paper address subitem 20-i? *

Copy and paste relevant sections from the manuscript (include quotes in quotation marks "like this" to indicate direct quotes from your manuscript), or elaborate on this item by providing additional information not in the ms, or briefly explain why the item is not applicable/relevant for your study

Because there was a little data missing, we conducted a completer analysis (vs intention-to-treat analysis).
Our sample was quite demographically homogeneous with the majority of women being partnered and well educated, as well as being born in Canada. However, our findings suggest that some subgroups of women may perceive mental health screening as more vulnerable. Future research should explore such women's views of mental health screening in greater depth.

21) Generalisability (external validity, applicability) of the trial findings

NPT: External validity of the trial findings according to the intervention, comparators, patients, and care providers or centers involved in the trial

21-i) Generalizability to other populations

Generalizability to other populations: In particular, discuss generalizability to a general Internet population, outside of a RCT setting, and general patient population, including applicability of the study results for other organizations

	1	2	3	4	5	
subitem not at all important	(•)	( )	( )	( )	( )	essential

Does your paper address subitem 21-i?

Copy and paste relevant sections from the manuscript (include quotes in quotation marks "like this" to indicate direct quotes from your manuscript), or elaborate on this item by providing additional information not in the ms, or briefly explain why the item is not applicable/relevant for your study

Does not address the generalizability to other populations as yet but focuses on pregnant demographic.

21-ii) Discuss if there were elements in the RCT that would be different in a routine application setting

Discuss if there were elements in the RCT that would be different in a routine application setting (e.g., prompts/reminders, more human involvement, training sessions or other co-interventions) and what impact the omission of these elements could have on use, adoption, or outcomes if the intervention is applied outside of a RCT setting.

	1	2	3	4	5	
subitem not at all important	○	○	○	○	◉	essential

Does your paper address subitem 21-ii?

Copy and paste relevant sections from the manuscript (include quotes in quotation marks "like this" to indicate direct quotes from your manuscript), or elaborate on this item by providing additional information not in the ms, or briefly explain why the item is not applicable/relevant for your study

Screening could be used in very similar way in primary health care as in RCT: screening link could be sent by email or text for completion of screening tools and results could be then sent to primary care offices.

OTHER INFORMATION

23) Registration number and name of trial registry

Does your paper address CONSORT subitem 23? *

Copy and paste relevant sections from the manuscript (include quotes in quotation marks "like this" to indicate direct quotes from your manuscript), or elaborate on this item by providing additional information not in the ms, or briefly explain why the item is not applicable/relevant for your study

Trial Registration: Clinicaltrials.gov NCT01899534; https://clinicaltrials.gov/ct2/show/NCT01899534 (Archived by WebCite at http://www.webcitation.org/6tRKtGC4M)

24) Where the full trial protocol can be accessed, if available

Does your paper address CONSORT subitem 24? *

Cite a Multimedia Appendix, other reference, or copy and paste relevant sections from the manuscript (include quotes in quotation marks "like this" to indicate direct quotes from your manuscript), or elaborate on this item by providing additional information not in the ms, or briefly explain why the item is not applicable/relevant for your study

Trial Registration: Clinicaltrials.gov NCT01899534; https://clinicaltrials.gov/ct2/show/NCT01899534 (Archived by WebCite at http://www.webcitation.org/6tRKtGC4M)

25) Sources of funding and other support (such as supply of drugs), role of funders

Does your paper address CONSORT subitem 25? *

Copy and paste relevant sections from the manuscript (include quotes in quotation marks "like this" to indicate direct quotes from your manuscript), or elaborate on this item by providing additional information not in the ms, or briefly explain why the item is not applicable/relevant for your study

Canadian Institute of Health Research CIHR-national health funder of Canada

X27) Conflicts of Interest (not a CONSORT item)

X27-i) State the relation of the study team towards the system being evaluated

In addition to the usual declaration of interests (financial or otherwise), also state the relation of the study team towards the system being evaluated, i.e., state if the authors/evaluators are distinct from or identical with the developers/sponsors of the intervention.

	1	2	3	4	5	
subitem not at all important	○	○	○	○	○	essential

Does your paper address subitem X27-i?

Copy and paste relevant sections from the manuscript (include quotes in quotation marks "like this" to indicate direct quotes from your manuscript), or elaborate on this item by providing additional information not in the ms, or briefly explain why the item is not applicable/relevant for your study

No conflicts of interest exist.

About the CONSORT EHEALTH checklist

As a result of using this checklist, did you make changes in your manuscript? *

- ( ) yes, major changes
- ( ) yes, minor changes
- (•) no

What were the most important changes you made as a result of using this checklist?

How much time did you spend on going through the checklist INCLUDING making changes in your manuscript *

8 hours

As a result of using this checklist, do you think your manuscript has improved? *

- ( ) yes
- ( ) no
- (•) Other: future articles will benefit

Would you like to become involved in the CONSORT EHEALTH group?

This would involve for example becoming involved in participating in a workshop and writing an "Explanation and Elaboration" document

- ( ) yes
- (•) no
- ( ) Other:

Any other comments or questions on CONSORT EHEALTH

I cannot possibly finish all of these fields in one sitting. Can I save it online and come back to it without losing all my data?
I could not submit this form because the document indicated my answers were too long; however, it did not indicate which fields were too long. I have tried to answer your questions thoroughly, but cannot then go back through the many fields and try to 'guess' which fields are too long. I will upload this as a pdf into the Journal's supplementary files, but I cannot submit this using this form. If you have another solution to [illegible]

STOP - Save this form as PDF before you click submit

To generate a record that you filled in this form, we recommend to generate a PDF of this page (on a Mac, simply select "print" and then select "print as PDF") before you submit it.

When you submit your (revised) paper to JMIR, please upload the PDF as supplementary file.

Don't worry if some text in the textboxes is cut off, as we still have the complete information in our database. Thank you!

Final step: Click submit !

Click submit so we have your answers in our database!

Submit

Never submit passwords through Google Forms.

Powered by

This form was created outside of your domain.
Report Abuse - Terms of Service - Additional Terms
